# Revisiting the Mechanisms of Immune Evasion Employed by Human Parasites

**DOI:** 10.3389/fcimb.2021.702125

**Published:** 2021-07-29

**Authors:** Monrat Chulanetra, Wanpen Chaicumpa

**Affiliations:** Center of Research Excellence on Therapeutic Proteins and Antibody Engineering, Department of Parasitology, Faculty of Medicine Siriraj Hospital, Mahidol University, Bangkok, Thailand

**Keywords:** parasite immune evasion, immunological privilege sites, antigenic disguise, antigenic mimicry, alternatively activated macrophages, immune checkpoints, regulatory immune cells, insect vectors

## Abstract

For the establishment of a successful infection, *i.e.*, long-term parasitism and a complete life cycle, parasites use various diverse mechanisms and factors, which they may be inherently bestowed with, or may acquire from the natural vector biting the host at the infection prelude, or may take over from the infecting host, to outmaneuver, evade, overcome, and/or suppress the host immunity, both innately and adaptively. This narrative review summarizes the up-to-date strategies exploited by a number of representative human parasites (protozoa and helminths) to counteract the target host immune defense. The revisited information should be useful for designing diagnostics and therapeutics as well as vaccines against the respective parasitic infections.

## Introduction

Parasites are eukaryotic organisms that live in or on host organisms and use the host to thrive. They may be single-celled (protozoa) or multicellular (metazoa) organisms. Parasites that cause diseases in humans can be divided into three main types: protozoa and helminths that live in the hosts (gut, lymphatic, blood circulation, cells, organs, and/or tissues) and ectoparasites (arthropods), such as hematophagous flies, mosquitoes, lice, bugs, fleas, and ticks, that feed on the blood or other products of the host. Protozoan parasites are classified in the kingdom Protista, while the helminths and arthropods belong to the kingdom Animalia. Helminthic parasites can be divided further into two phyla: Nematoda (Nemathelminthes, nematodes, or roundworms) and Platyhelminthes (trematodes or flukes and cestodes or flatworms). **Table 1** gives some examples of protozoa and helminths that cause human diseases.

**Table 1:** Examples of protozoa and helminths that cause diseases in humans.

Protozoa	Helminths (Metazoa)		
	Nematode	Platyhelminth	
		Cestode (Flat worm)	Trematode (Fluke)
*Acanthamoeba* spp. *Babesia bigemina* *Balamuthia mandrillaris* *Cryptosporidium parvum* *Cyclospora cayetanensis* *Entomoeba histolytica* *Giardia lamblia* *Leishmania* spp. *Naegleria fowleri* *Neobalantidium coli (formerly Balantidium coli*) *Plasmodium* spp. *Sappinia pedata* *Tetratrichomonas empyemagena* *Thelazia gulosa* *Toxoplasma gondii* *Trichomonas vaginalis* *Trypanosoma* spp.	*Ancylostoma duodenale* *Angiostrongylus cantonensis* *Ascaris lumbricoides* *Brugia malayi* *B. timori* *Clonorchis sinensis* *Dracunculus medinensis* *Enterobius vermicularis* *Gnathostoma spinigerum* *Loa loa* *Necator americanus* *Oncocerca volvulus* *Strongyloides stercoralis* *Trichinella* spp. *Wuchereria bancrofti*	*Dibothriocephalus latus (formerly Diphyllobothrium latum*) *Echinococcus granulosus* *Hymenolepis nana* *Taenia solium* *T. saginata*	*Clonorchis sinensis* *Fasciola gigantiga* *F. hepatica* *Opisthorchis felineus* *O. viverrini* *Paragonimus* spp. *Schistosoma* spp.

Although the parasites are classified in diverse taxonomic families based on their distinct morphology and molecular attributes, they are endowed with and share the same capacity to overcome, exploit, evade, modulate, and/or downregulate the immune responses of the infected host in order to maintain their parasitism, *i.e.*, long-term survival and completion of their life cycle. This review article summarizes the most important strategies used by representative protozoa and helminths to overcome, evade, resist, suppress, and/or manipulate the host immune apparatus/mechanisms that are directed against them. The gathered information could assist designing diagnostics, novel treatment biologics/regimens, and vaccines to counteract the diseases caused by the respective parasites.

## How Parasites Overcome the Hosts’ Physical and Physiological Barriers

Usually intact skin is a formidable barrier that acts as the first line of defense against microorganisms that may try to gain entry into a host. Nevertheless, many helminthic parasites can penetrate directly through human skin to establish an infection. The mature larvae of hookworms (*Ancylostoma duodenale* and *Necator americanus*) and the filariform larvae of *Strongyloides stercoralis* (threadworm), for example, can penetrate the skin of humans who walk barefoot on contaminated soil. The larvae cause a focal itchy area (ground itch) and rashes and sometimes papules at the worm-penetrating site. The larvae of *A. duodenale* can infect humans who ingest contaminated matter. The skin-penetrating hookworm larvae may also cause cutaneous larva migrans or a creeping eruption, which appears as a snake-like track (Beaver, 1956; Albanese et al., 2001). Mammalian host skin and its skin secretions are particularly abundant in urocanic acid, a histidine metabolite that attracts the filariform larvae of *Strongyloides* spp. (Safer et al., 2007). The cercariae of the human *Schistosoma* spp. (blood fluke), which can emerge from certain types of snail intermediate hosts and can swim around in freshwater, can penetrate the skin of humans who they come into contact with. Inside the mammalian host, these infective larvae further develop into tail-less schistosomulae and adult parasites that cause different forms of schistosomiasis (Bilharziasis), depending on the species of the infecting flukes. *Schistosoma haematobium* causes urogenital schistosomiasis, which could turn into bladder cancer (Ajibola et al., 2019), while *S. japonicum*, *S. mansoni, S. mekongi, S. guineensis*, and *S. intercalatum* cause hepatic and intestinal schistosomiasis (Elbaz and Esmat, 2013). The cercariae of bird and animal schistosomes can also penetrate human skin, but do not develop further; rather, they are retained at the penetration site and cause cercarial dermatitis (swimmer’s itch, clam-digger’s itch, duck itch) (Kolářová, 2007; Horák et al., 2015). Other parasites, like *Plasmodium* spp., kinetoplastids/trypanosomatids (*Trypanosoma* spp., *Leishmania* spp.), *Babesia*, and filarial worms (*Brugia malayi*, *B. pahangi*, *B. timori*, *Wuchereria bancrofti, Oncocerca volvulus, Loa loa*), require vectors, such as mosquitoes, hematophagous flies, bugs, or ticks, to deliver their infective stage into the human host.

A bite by a *Plasmodium-*infected *Anopheles* mosquito can transmit around 50–200 *Plasmodium* sporozoites (pre-erythrocytic stage, PE) into the human skin (Beier et al., 1991; Gomes et al., 2016). The biting mosquitoes not only transmit the PE, but also release many different salivary components into the skin, such as anti-histamines, vasodilators, anti-coagulants, platelet aggregation inhibitors, and immunomodulators, which help the sporozoites’ survival (Zheng et al., 2014). Although many of the *Plasmodium* sporozoites are destroyed at the inoculation site by the host’s local innate defense factors, some of them can manage to avoid the immunity’s defense mechanisms by various mechanical strategies, including rapid extracellular gliding motility (Vanderberg, 1974), cell-traversal motility (traverse within the transient vacuoles of host cells, including immune cells) to exit the skin by invading the vasculatures and lymphatics (Mota et al., 1999), and invasion motility to infect hepatocytes, where they generate the erythrocyte-infecting form, *i.e*., the merozoites (Yuda and Ishino, 2004; Tavares et al., 2013; Risco-Castillo et al., 2015; Gomes et al., 2016). A protein named thrombospondin-related anonymous protein (TRAP), found on the surface of the *Plasmodium* sporozoites and their micronemes (secretory organelles located on the apical cytoplasm of protozoa), helps them to interact with the host surface molecules, providing them with gliding motility to exit the dermis through the blood vessels (Müller et al., 1993; Gomes et al., 2016). The sporozoites leave the blood circulation in the liver by crossing the liver sinusoidal cell layer to infect the hepatocytes. During the cell traversal, the sporozoites use perforin-like protein (PLP1) to escape degradation by the host cell’s lysosomes, and then migrate to the liver where they egress (Patarroyo et al., 2011). TRAP may also play a role in sporozoite invasion of the hepatocytes by binding to sulfated glycoconjugate motifs on the liver cells (Patarroyo et al., 2011). Circumsporozoite protein (CSP), which is the sporozoites’ surface coat, binds to the particularly highly sulfated glycosaminoglycan chains in liver heparan sulfate proteoglycans (HSPGs) produced by the hepatocytes and stellate cells (Ellis et al., 1983; Pradel et al., 2002; Ménard et al., 2013). Within the invaded hepatocytes, the sporozoites reside in parasitophorous vacuoles (PVs), where they generate an enormous number of blood-stage merozoites.

The *Trypanosoma brucei* subspecies *rhodesiense* and *gambiense*, the causative agents of African trypanosomiasis or sleeping sickness, have the obligate hematophagous *Glossina* tsetse fly as the vector to deliver their infective metacyclic stage (short-form trypomastigotes) into human skin during a fly bite applied for blood-meal taking. The saliva of the tsetse fly not only contains anti-hemostatic compounds for efficient blood feeding, but also other complex components that help the parasite to establish a successful infection by manipulating the human skin microenvironment into a trypanosome-appreciative habitat. The tsetse fly saliva contains the thromboregulatory compounds 5′-nucleotidase-related apyrase and adenosine deaminase, which inhibit blood coagulation and platelet aggregation at the fly-bite site (Caljon et al., 2010). The fly saliva also contains an allergen named Antigen 5 (a homolog of antigen 5 allergens of Hymenoptera venoms) that can cause type-1 hypersensitivity by activating IgE-sensitized mast cells to degranulate and release vasoactive mediators. These mediators cause the vasodilatation and extravasation of immune cells, myeloid phagocytic cells, antibodies, and complement factors (C3a, C5a), leading to inflammation, *i.e*., increased vascular permeability and immune cell recruitment and activation (Caljon et al., 2009). Gloss 2 peptide (DTAFSCHFFEIYLSNCFNKEKYIKNYLQIM) contained in the inoculated tsetse fly saliva antagonizes the host immune response by inhibiting the secretion of tumor necrosis factor-α (TNF-α), interferon-gamma (IFN-γ), interleukin (IL)-6, and IL-10 (immunosuppressive cytokine) from the host’s immune cells. These immune suppressions facilitate the fly’s blood feeding and the trypanosomes transmission; thus helping establishment of the trypanosome infection (Bai et al., 2015). Besides, a *T. brucei* protein named kinesin heavy chain 1 (TbKHC1) binds to the C-type lectin receptor (SIGN-R1) to trigger the release of IL-10 and arginase-1 from myeloid cells (alternatively activated macrophages or M2); both these proteins can suppress the inflammatory response induced at the fly-bite site, which promotes the trypanosome growth and its settlement in the host in the early infective stage (De Muylder et al., 2013). Moreover, at the late stage of the infection, the TbKHC1 inhibits nitric oxide (NO) synthesis, which contributes to liver pathogenicity (De Muylder et al., 2013). The trypanosomes first express the variant surface glycoproteins (VSGs) in the tsetse fly vector, at the metacyclic stage, in preparation for transfer into the mammal and survive within the blood circulation of the mammalian host (Graham and Barry, 1995). Usually, human serum contains two high-density lipoproteins (HDL) termed trypanosome lytic factors (TLFs), namely TLF1 and TLF2, which represent hemoglobin-binding proteins, haptoglobin-related proteins (Hpr), and a pore-forming protein called apolipoprotein L-1 (apoL-1) that are toxic to the trypanosomes (Thomson et al., 2009). Nevertheless, *T. brucei* have evolved resistance to the TLFs by secreting serum resistant-associated protein (SRA), which can block the activity of the TLFs. Thereby, they can further develop to cause sleeping sickness, which manifests as neuropsychiatric symptoms, including sleep disruption, confusion, lethargy, and convulsions. The disease can be fatal, if not treated.

The hematophagous assassin (triatomine) bugs of the family Reduviidae, subfamily Triatominae, order Hemiptera, are vectors that transmit *Trypanosoma cruzi*, the causative agent of Chagas disease or American trypanosomiasis, to humans. The triatomine bugs prefer to bite the human face, particularly around the mouth and eyes (they are colloquially called kissing bugs) (Mekonnen; Moncayo and Yanine, 2006). While taking the blood meal on the face of the sleeping human host or at the end of a blood meal, the bug defecates, whereby the infective trypanosomes contained in the feces are mechanically passed onto the host skin near to the bite wound (Moncayo and Yanine, 2006). The irritation and itching from the insect bite cause the host to scratch or rub the bite area; thus facilitating entry of the infective parasite into the bite wound, or the ocular and the oral mucous membranes, if the bite site is near to the eyes and lips.

Intestinal parasites that infect human hosts *via* the oral route have to overcome the host’s gastric acidity; also, they must penetrate the intestinal mucus and glycocalices before reaching the enterocytes underneath to establish infection. Ingested cysts of *Giardia lamblia* (synonym *G. intestinalis, G. duodenalis*) and *Entamoeba histolytica* can withstand the low pH in the stomach and can thereby gain accessibility to the lower intestinal tract, where the trophozoites can germinate and colonize the host. *G. lamblia* colonizes the duodenum, jejunum, and ileum, and lives on nutrients and amino acids (especially arginine) from the intestinal fluids, causing persistent diarrhea in humans, called giardiasis (Schofield et al., 1992; Hemphill et al., 2019). The *G. lamblia* trophozoites produce an arginine-consuming enzyme, namely arginine deiminase (ADI), for active consumption of the amino acid arginine as the main energy source (Stadelmann et al., 2012). Arginine deprivation caused by the parasite’s ADI leads to cycle arrest of the intestinal epithelial cells through the reduced polyamine levels and high expression of mRNA of the cell cycle regulatory proteins BTG3 and GADD45A*;* hence, this slows down the epithelial cell turnover, which in turn effectively helps the pathogen to maintain a more stable niche for colonization (Stadelmann et al., 2012). The intestinal epithelium disturbance results in increased mucosal permeability and diarrhea in the host (Stadelmann et al., 2012). Ingested oocysts containing invasive sporozoites of *Cryptosporidium parvum* also pass through the gastric acidic environment and enter the small intestinal epithelial cells, where they thrive in the parasite induced-parasitophorous vacuoles (PVs) at the apical part of the enterocytes just underneath the brush border. *Cryptosporidium parvum* causes severe diarrhea, called cryptosporidiosis, particularly in children and immunocompromised subjects (Striepen, 2013; Hemphill et al., 2019). *Entamoeba histolytica* trophozoites use cysteine proteases and glycosidases to cleave MUC2 mucin to breach the mucus layer and colonize the colon by using their surface lectin to bind with high affinity to mucin, causing intestinal amoebiasis (Moncada et al., 2003; Lidell et al., 2006; García et al., 2015; Nakada-Tsukui and Nozaki, 2016). *Entamoeba histolytica* trophozoites may invade the colon wall by digesting extracellular matrices (Nakada-Tsukui and Nozaki, 2016) and may then be disseminated to other organs (extraintestinal amoebiasis), mainly the liver and brain; thereby causing life-threatening liver and brain abscess, respectively (Savanat and Chaicumpa, 1969; Hemphill et al., 2019). *Echinococcus granulosus* exploits the bile acid and salt in the duodenum to stimulate the hatching of its ingested ova to oncospheres in the small intestine, which later pass through the portal and lymphatic vessels to the liver, where they usually develop into hydatid cysts. Less frequently, the *E. granulosus* onchospheres may also reach the lungs, brain, bones, or any other organ of the human host and form hydatid cysts therein (Wen et al., 2019).

## Parasites Are Sequestered in the Host’s Immunological Privileged Sites

Many parasites evade the host immunity by residing in anatomical sites that are devoid of the host immunological apparatus/factors, called immunological privileged sites, such as inside the host cells or hollow organs. Red blood cells infected with the erythrocytic stage (merozoites) of the *Plasmodium* spp. (malaria-causing parasite) form rosettes with an uninfected counterpart to escape the host’s antibodies and complements (Moll et al., 2015). Since human red blood cells are devoid of major histocompatibility complex (MHC) molecules, both class I and class II, the merozoites also escape from the MHC-restricted cytotoxic CD8^+^ lymphocyte killing (Bowen and Walker, 2005). *Plasmodium falciparum* merozoites in the infected erythrocytes mature to the ring stage, trophozoites, and schizonts. Only the young form (ring stage) is found in the blood circulation, while the more mature forms (trophozoites and schizonts) disappear from the blood circulation through a phenomenon called “sequestration” (standing apart), which is another mechanism of the malaria parasite for immune evasion. The *Plasmodium* sequestration is mediated by variant surface antigens encoded by *var* genes named *P. falciparum* erythrocyte membrane proteins (PfEMPs). The PfEMPs tether the parasitized erythrocytes to CD36 [other names: platelet glycoprotein 4, fatty acid translocase (FAT), scavenger receptor class B member 3 (SCARB3), glycoproteins 88 (GP), GPIIIb (GPIIIB), or GPIV (GPIV)], and thrombospondin receptors on the microvascular endothelium and other host cells (cytoadherence) in many organs. Thus, the infected red blood cells can avoid circulating to the spleen and hence can escape splenic destruction, and subsequently they can cause high parasitemia (Miller, 1965). One study found that a splenectomized squirrel monkey had a reduced sequestration of *Plasmodium*-infected erythrocytes compared to an intact animal (David et al., 1983). Antibodies could inhibit and reverse the infected erythrocytes binding to the host cells *in vitro*, and a passive transfer of immune serum into an infected intact animal could reverse the *in vivo* sequestration (David et al., 1983). *Babesia* spp. invade red blood cells and also induce adhesion molecules on the red blood cell surface, which then cause adherence of the parasite-infected erythrocytes to the vascular endothelium similar to the *P. falciparum* (Allred and Al-Khedery, 2004; Bloch et al., 2019). As a result, the parasites can similarly evade elimination by the spleen and complete their life cycle by infecting new erythrocytes to perpetuate the infection.

In its a steady state, skin is immunocompromised by the activity of localized-natural CD25^+^Foxp3^+^ regulatory T cells (nTregs), which have the function to suppress unwanted immune responses to antigens/agents to which the skin is regularly exposed (Peters and Sacks, 2006). The immunocompromised human skin is a privileged site for the survival of *Leishmania*-infective metacyclic promastigotes that are introduced into the host skin during a bite by the infected sand fly vector. The *Leishmania* promastigotes are then ingested by macrophages and transformed into amastigotes, whereby they are unreachable by the antibody and complement proteins. *Leishmania* amastigotes survive and multiply inside the phagocytes by resisting an array of the host intracellular microbicidal factors to establish chronic infection (Wright and Silverstein, 1983; Da Silva et al., 1989; Gupta et al., 2013).

The infectious (L3) larvae of *Trichinella spiralis* and other *Trichinella* species, including *T. britovi, T. nelsoni*, and *T. nativa*, form nurse cells in the infected muscle cells of the host. The nurse cells protect the parasites from the host immune recognition and also provide them with host-acquired nutrients (Wu et al., 2008).

Many parasites, *e.g*., *Taenia* spp., *Ascaris lumbricoides*, and *Opisthorchis viverrini*, live in the hollow organs of the host, such as in the intestinal lumen and bile duct. While in these sites, they cannot be reached by the effective serum IgM and IgG antibodies, whereas the locally produced secretory IgA cannot activate the complement. The intestinal epithelium also produces a number of factors that can potentially inactivate complement proteins, including intestinal trefoil factor (ITF), a small protease-resistant peptide produced and secreted onto the mucosal surface by goblet cells, and decay accelerating factor (DAF), a protein produced by columnar epithelium that regulates complement activation (Sun et al., 1999; Andoh et al., 2001). The intestinal lumen and the mucosal surface are also rich sources of nutrients.

## Antigenic Disguise

Several infecting parasites cover themselves with the host/host-like factors, *e.g*., proteins or glycoconjugates, so that they will not be recognized as foreign substances by the host immune system. Newly formed *Plasmodium* merozoites that exit from hepatocytes unavoidably encounter phagocytic cells in the liver, including Kupffer cells and dendritic cells. To avoid being killed by these phagocytes, the merozoites hide themselves within the merosomes derived from infected hepatocytes while they egress (Sturn et al., 2006; Melanie et al., 2019). Schistosomulae and adult *S. mansoni* acquire host antigens, including erythrocyte antigens (Rhesus, M, N, S, and Duffy), complement proteins, integrins, CD44, collagen, immunoglobulins, MHC class-I, and β_2-_microglobulin, on their surface to avoid host immune recognition (Clegg et al., 1971; Goldring et al., 1976; Kemp et al., 1977; Snary et al., 1980; Braschi et al., 2006; Braschi and Wilson, 2006). *Schistosoma japonicum* and *S. mansoni* produce surface-exposed paramyosin (a 94/97 kDa muscular protein, *i.e*., Sj97 and Sm97) on the surface that binds to the host Fc fragments of immunoglobulins and complement C1 and C9 proteins for antigenic camouflage and anti-complement activity (Laclette et al., 1992; Loukas et al., 2001; Deng et al., 2007). The microfilariae of *Onchocerca volvulus* (filarial parasites causing river blindness) decorate themselves with Factor H, which is the main regulator of the complement alternative pathway that they sequestered from the host blood to conceal their surface antigens and avoid complement attack (Meri et al., 2002). *Trypanosoma cruzi* trans-sialidases transfer sialic acid from the host glycoconjugates to their surface to subvert the host immune system; hence favoring the parasite survival and the establishment of chronic infection (Nardy et al., 2016). The outermost layer (pericyst) that encases the *Echinococcus granulosus* hydatid cyst formed in the host tissues (such as the liver and lung) is made of the host cells. Therefore, the pericyst not only plays an important role in the parasite development and survival, but also in the host immune evasion through the antigenic camouflage mechanism (Golzari and Sokouti, 2014).

## Parasites Exist in Different Developmental Forms

Most parasites have a complex biology, including different developmental stages or variants, which express different antigenic profiles; thus implicitly inducing the host to express multifaceted stage/variant-specific immune responses. The host immune response to one antigenic set of a parasite stage or variant is usually ineffective against the others.

The excretory–secretory products of *Brugia malayi* microfilariae and adult male and female parasites have different protein profiles as revealed by proteomic analysis (Moreno and Geary, 2008). Among the 228 proteins identified, only 32 proteins are shared by the microfilariae and the two adult parasite genders (Moreno and Geary, 2008). The different profiles of the proteins among the larvae and the adult males and females may reflect their different biological processes as well as their different strategies for host immune evasion (Moreno and Geary, 2008; Reamtong et al., 2019). The life cycle of *Schistosoma* species is relatively complex and involves stages in the invertebrate intermediate host, free-living, and in the definitive vertebrate host, including ova, miracidia, sporocysts, cercariae, schistosomulae, and adult males and females. After penetrating the skin of the definitive (vertebrate) host, the infective cercariae lose their bifurcated tails and transform into schistosomulae, which enter the vasculature and mature into adult males and females during their trans-pulmonary journey to the tissues of their species-specific destinations (mesenteric venous plexus in the case of *S. japonicum*, *S. mansoni*, and *S. mekongi*, and the urinary tract and bladder for *S. haematobium*). The fully mature male and female worms copulate and the females produce eggs, which induce an immune-mediated granulomatous response causing local and systemic pathological manifestations (*S. haematobium* can cause bladder cancer) (Khurana et al., 2005). While in the mammalian host, the *Schistosoma* parasites express stage-specific regulatory proteins and glycans that are molecularly different (Gryseels et al., 2006; Fitzpatrick et al., 2009; Colley et al., 2014; Smit et al., 2015). It is believed that the specific antigen expression by the different developmental stages could be the parasite’s strategy to escape the host immune system (McWilliam et al., 2013). The secreted egg antigens of *S. haematobium* can stimulate an antibody response, but the antibodies are short-lived (also short-lived memory B-cell response) and non-protective against the homologous re-infection (Mitchell et al., 2012).

The trypanosome parasite has different morphological forms, including metacyclic promastigotes in the vector, amastigotes in mammalian tissue cells, and trypomastigotes in the host blood, all of which are antigenically distinct. Likewise, the malaria parasite at its different stages (sporozoites, merozoites/schizonts, trophozoites, and gametocytes) expresses functionally different proteins, as revealed in one study by using a throughput proteomics approach (Florens et al., 2002).

## Antigenic Variation

Antigenic variation is an important strategy used by several blood-borne protozoan parasites for their survival in immunocompetent hosts. Trypanosome infection causes waves of parasitemia, *i.e*., the number of the trypomastigotes in the blood rises and falls. The blood-stage trypomastigotes express highly immunogenic antigens called variant-specific glycoproteins (VSGs) on their surface. The VSG proteins (60 kDa) are densely packed and form a surface coating about 12–15 nm thick on the trypomastigotes (Cross, 1975; Pays, 2006). The VSGs enable the trypomastigotes to evade the mammalian host’s immune system by extensive antigenic variation. Trypomastigotes obtained from successive peaks of parasitemia express antigenically distinct VSGs. The robust antibodies produced in response to the highly immunogenic VSG on the surface of one trypomastigote variant can eliminate most of the homologous parasitic variants by either complement-mediated lysis or phagocytosis, in which the antibodies function as opsonin (Cross, 1975; Umekita et al., 1988; Umekita et al., 1997; Pays, 2006). At the same time, however, there is a selection of some trypomastigotes that express another set of VSGs to which the antibodies to the former variant are non-cross reactive, and thus are ineffective against the newly emerged heterologous variant (Pays, 2006). Such VSG switching is a natural ability of the trypomastigotes and is inherent in them through the existence of a thousand VSG genes at the subtelomeric region of the main chromosome, mini-chromosomes, and at the expression sites (located in a discrete extra-nucleolar body that contains the transcription and RNA processing machinery necessary for the high-level expression of VSG genes). Besides, the VSG genes are capable of making chimeric new ones. Thus, the potential for surface variation during the trypanosomal protracted infection is endless (Rothwell et al., 1985; Rudenko et al., 1996; Navarro and Gull, 2001). The switching mechanisms of the VSGs involve either *in situ* activation, reciprocal telomere exchange, or gene conversion (Rudenko et al., 1996). Relapsed variants of *Plasmodium* and *Babesia*, have antigens that are usually different from the parental parasites (Cox, 1962; Recker et al., 2011; Bloch et al., 2019).

## Antigen Capping

Many parasites dampen the host humoral response directed to their surface antigens by allowing the antibodies to bind to their surface antigen; then, they rapidly get rid of the immune complexes either by internalization *via* the endocytic process followed by destruction of the antibody in the lysosome, or by stripping off the immune complexes from their surface.

Besides the antigenic variation, trypomastigotes of *T. brucei* possess a highly active endocytotic system, which they use as an additional mechanism for the removal of surface-bound antibodies (antibody clearance) (Pal et al., 2003; Marquez-Contreras, 2018). The trypanosome redistributes the immune complexes formed by the host antibodies that are bound to the VSGs on their surface to their flagella pockets (Engstler et al., 2004). This parasite-mediated process is known as “Capping” (Barry, 1979). The endosomally localized RAB5 and RAB11 proteins of the trypanosome then mediate endocytosis of the immune complexes, whereby the antibodies in the complexes are subsequently destroyed by proteolysis within the *T. brucei* recycling system (Pal et al., 2003). Trophozoites of the invasive *E. histolytica*, not of the non-invasive *E. dispar*, can redistribute surface immune complexes formed by RAB11 and RAB11 antibodies, and generate caps at the posterior pole of the parasite. The capped-immune complexes may be followed either by endocytosis and degradation, as for *T. brucei* antigen capping, or by gradually forming a constriction ring and stripping off the capped-immune complexes (Espinosa-Cantellano and Martínez-Palomo, 2000; Chávez-Munguía et al., 2012). Binding of the host antibody to *Leishmania* amastigotes causes the parasite surface membrane antigens to aggregate, move along the longitudinal cell axis, form polar cell caps, and subsequently disappear (Dwyer, 1976). Also, in one study, antibody-exposed promastigotes showed tripartite membrane antigen capping, including the major anterior cell pole cap, and minor caps at the posterior cell pole and flagella tip region, which later disappeared (Dwyer, 1976). A few hours later, the parasite membrane antigens removed by capping were regenerated and detectable at the cell surface (Dwyer, 1976).

## Molecular Mimicry (Sharing of Antigens Between the Parasite and Host)

Molecular mimicry refers to the situation where a foreign antigen shares a sequence or structural similarities with the host’s self-antigens. Many parasites produce antigens that have a molecular structure that resembles the host components (Damian, 1964). Being recognized as “Self”, the parasites protect themselves from the host immune system by maintaining the host immune steady state.

*Schistosoma mansoni* and *S. haematobium* possess a complement C2 receptor inhibitory trispannin (CRIT) gene that shares 98% identical nucleotides with its human ortholog (Inal, 1999). The free-living cercariae of the blood flukes are complement sensitive, but after skin penetration, they transform into schistosomulae, which express a high level of CRIT, and thus are complement resistant (Deng et al., 2003). *Schistosoma* eggs also express a high level of CRIT (Deng et al., 2003). *Brugia malayi* generates a protein that resembles keratinocytes periphilin-1 protein (potentially involved in epithelial differentiation and the maintenance of epidermal integrity for skin formidability) (Kazerounian and Aho, 2003). *Plasmodium falciparum* erythrocyte membrane protein 1 (PfEMP1) has a 14 amino acid motif that is identical to part of the heparin-binding domain of the host vitronectin (a 70 kDa glycoprotein in serum and tissues that is involved in cell adhesion and spreading) and that functions as an inhibitor of the membrane-damaging effect of the complement membrane attack complex (MAC) by binding with C5b67, C5b-8, and C5b-9 to inhibit MAC membrane insertion (Ludin et al., 2011). Many parasites produce human blood group-like antigens. Blood group A, B, and O antigens were found to be present in *S. japonicum* (Matsuse, 1956). *Fasciola hepatica* produces active substances like human blood A, H, and Lewis (Le) antigens (Ben-Ismail et al., 1982). *Ascaris lumbricoides* var. *suum* and hookworms (*Necator americanus* and *Ancylostoma duodenale*) possess A- and B-like blood group antigens in their polysaccharides (Oliver-Gonzalez, 1944; Ota et al., 1954; Ota and Tadokoro, 1954). *Trichinella spiralis* produces Forssman antigen (Mauss, 1941). A blood group P1 substance was found in hydatid cyst fluid of *E. granulosus* (Levine et al., 1958). *Plasmodium falciparum* circumsporozoite protein (CSP) has a motif that is nearly identical to the cytoadhesive region of mammalian thrombospondin (Robson et al., 1988). *Trypanosoma cruzi* produces proteins that mimic myocardial cell proteins and induce autoimmunity causing Chagasic cardiomyopathy (Chagas disease—named after the Brazilian physician Carlos Chagas who discovered *T. cruzi* and the disease) (Tanowitz et al., 2009).

## Parasites Modify and/or Suppress the Host Immunological Factors/Immune Responses

### Digestion of the Host Matrices, Antimicrobial Peptides, Antibodies, and/or Inhibition of the Host Factors

Both secreted and membrane-bound cysteine proteases of *E. histolytica* trophozoites digest extracellular matrix proteins, fibronectin, and laminin for successful invasion of the host tissues. *E. histolytica* uses cysteine proteases to circumvent the host innate and specific humoral defense factors through the cleavage of intestinal antimicrobial peptides, *e.g*., cathelicidins and secretory IgA and serum IgG antibodies (the morphologically identical *E. dispar* lacks these virulent properties and is non-pathogenic) (Que and Reed, 2000). Cruzipain, the main cysteine protease of *T. cruzi*, can digest all human IgG subclasses at the hinge region to yield either Fab fragments from IgG1 and IgG3, or F(ab)′_2_ fragments from IgG2 and IgG4, and also Fc fragments (Berasain et al., 2003). The enzyme also was found to cleave heavy chains of all IgG subclasses between the CH2 and CH3 domains to produce 14 kDa Fc-like fragments (Berasain et al., 2003). Schistosomulae of *S. mansoni* cleave IgG bound to their surface to yield Fab fragments by using pH and temperature-dependent trypsin-like endoprotease and metalloaminopeptidase (Auriault et al., 1981). Cercarial and schistosomular extracts of *S. mansoni* cleave human, mouse, and rat IgE (Pleass et al., 2000). *Schistosoma mansoni* produces the Kunitz-type protease inhibitor (SmKI-1), which inhibits mammalian trypsin, chymotrypsin, neutrophil elastase, FXa, and plasma kallikrein, which might be another parasite mechanism to evade the defense mechanisms of the mammalian hosts (Ranasinghe et al., 2015). SmKI-1 is found in the adult worm tegument and in excretory/secretory products, as well as in the sub-shell region of the eggs (Ranasinghe et al., 2015).

### How Parasites Resist Being Killed by the Host Cells

Parasites can manage to resist being killed by interfering with the phagocytic activity and by resisting the highly toxic oxidative radicals and digestive enzymes of the host cells in the environment where they thrive to complete their life cycle.

*Plasmodium* merozoites produce hemozoin (malaria pigment), which interferes with the phagocytic functions of macrophages, *i.e*., macrophages that are loaded with *Plasmodium* hemozoin cannot phagocytose more infected red blood cells, as well as have a reduced oxidative radical production (Belachew, 2018). *Leishmania infantum* and *L. major* express 3′-nucleotidase/nuclease (3′NT/NU) to digest neutrophil extracellular trap (NET) (Guimarães-Costa et al., 2014). Endonuclease (Lundep) present in sand fly saliva also helps the *Leishmania* spp. to survive in the initial infection phase (Chagas et al., 2014). Lipophosphoglycan (LPG), a glycolipid abundantly found on the surface of *L. donovani* promastigotes (the causative agent of visceral leishmaniasis or Kala-azar), resists NET-mediated killing and hinders the expression of the late endosomal markers LAMP1 and RAB7, which inhibits phagosome maturation (the fusion of phagosome with lysosome) by impairing recruitment of the lysosomal marker LAMP1 and protein kinase C-alpha (PKCα) to the phagosome. This allows the promastigotes to survive inside macrophages, to transform into amastigotes, and to multiply therein to establish chronic leishmaniasis (Chang and Dwyer, 1976; Holm et al., 2001; Gabriel et al., 2010). *Leishmania amazonensis* and *L. mexicana* form large parasitophorous vacuoles (PVs) that share features with the host’s late endosome/lysosome to dilute the detrimental effect of the oxidative radicals (Wilson et al., 2008). Kinetoplastid parasites of the family Trypanosomatidae discharge acid phosphatase (ACP) from a flagella pocket to dephosphorylate the parasitophorous vacuole membrane (PVM) and inhibit hydrogen peroxide production by the macrophages (Baghaei, 2003; Navabi and Soleimanifard, 2015). *Leishmania donovani* promastigotes inhibit phagosome maturation (phagolysosome biogenesis) by hindering the recruitment of synaptotagmin V to nascent phagosomes to exclude vesicular proton-ATPase; thus, the phagosome cannot be acidified (Vinet et al., 2009). *Leishmania donovani* chemotactic factor (LCF) inhibits polymorphonuclear cells generating inflammatory reactive oxygen species through a respiratory burst *via* the lipoxin A 4 (LPXA4) receptor named ALX/FPRL-1 (Wenzel and Van Zandbergen, 2009).

*Trypanosoma cruzi* phagocytosed by macrophages manage to survive in the highly oxidative intracellular environment by generating a complex network of anti-oxidant enzymes in the phagolysosome, including peroxidases (APX, CPX, and MPX), catalase, trypanothione reductase, and superoxide dismutases (SOD), which are distributed into various cellular compartments, including glycosomes, mitochondria, cytosol, and endoplasmic reticulum (ER). The anti-oxidant enzymes detoxify the detrimental reactive oxygen species (ROS): hydrogen peroxide (H_2_O_2_), nitric oxide (NO), and, particularly, the peroxynitrite (ONOO^−^), which produces secondary free radicals, such as nitrogen dioxide (NO_2_), carbonate $\left(\text{C}{\text{O}}_{3}^{-}\right)$, and hydroxyl radicals (OH^−^). These reactive oxygen species can otherwise cause the parasite damage and death within the phagolysosome (Finzi et al., 2004; Piñeyro et al., 2011). Trypanosome trans-sialidase transfers sialic acid from the lysosomal-associated membrane proteins (LAMPs), *i.e*., LAMP1 and LAMP2, to the parasite surface mucin-like glycoprotein, which favors cellular invasion of the parasite (Schenkman et al., 1992; Schenkman and Eichinger, 1993). Desialylation of the LAMPs also causes the phagosomal membrane to rupture and release the parasite from the phagolysosome to the cytosol, where it differentiates into replicative amastigotes. In the cytosol, ROS promotes the growth of *T. cruzi* amastigotes by facilitating the parasite accessibility to iron. The amastigotes then differentiate into bloodstream trypomastigotes (Cardoso et al., 2016).

*Toxoplasma gondii*, the obligate intracellular parasite of the phylum Apicomplexa (included also in the phylum are *Cryptosporidium* and *Plasmodium*) develops several strategies to avoid destruction by the host cells. The Apicomplexan parasites can penetrate the host cells directly by actin-based motility, called gliding motility (King, 1988). The *T. gondii* sporozoites from oocysts or bradyzoites from tissue cysts are a kind of Trojan horse that rapidly, quietly, and efficiently invades host cells by secreting proteins from their micronemes (MICs) and rhoptries (ROPs and RONs) for entering cells and for forming host-derived parasitophorous vacuoles (PVs), where they transform to tachyzoites and multiply therein by a process called endodyogeny (Delbac et al., 2001). The host cell-derived PV membrane (PVM) is permeable only for small molecules and nutrients acquired from the host and needed for the parasite growth (Delbac et al., 2001). The PVM’s selective permeability is mediated by *Toxoplasma*-secreted proteins, *i.e*., GRA17, which is conserved across PV-residing Apicomplexans, and GRA23, which is localized to the PVM and induces the passive transport of small molecules and nutrients across the PVM (Gold et al., 2015). The *T. gondii* GRA17 protein is related to the putative *Plasmodium* translocon protein named EXP2 (Garten et al., 2018). The *T. gondii* tachyzoites use micronemal proteins containing epidermal growth factor (EGF)-like domains, namely TgMICs, TgMIC3, and TgMIC6, to activate the EGF receptor to prevent binding of the host molecules to the PVM, by which they can avoid destruction by cellular autophagy (Meissner et al., 2002; Wang et al., 2009; Muniz-Feliciano et al., 2013). The newly generated tachyzoites in the PV can then egress to invade other host cells to perpetuate the infection. *Toxoplasma gondii* resist oxidative damage by using their cytosolic and mitochondrial superoxide dismutases, peroxiredoxins, glutathione-S-transferase, glutaredoxin, and catalase to decompose the reactive oxygen species (ROS) (Ding et al., 2004). Thioredoxin reductase of *T. gondii* maintains a thioredoxin-reduced state during NADPH consumption, which helps the parasite to resist the oxidative burst (Xue et al., 2017). *Toxoplasma* destroys iNOS to reduce nitric oxide production by activating the infecting macrophage to produce transforming growth factor (TGF)-β1, which acts in an autocrine fashion through the SMAD2 and SMAD3 proteins (signal transducers and transcriptional modulators), which are phosphorylated/activated by the signal from the TGF-β1 receptor. *T. gondii-*programmed cell death 5 (TgPDCD5) and dense granule antigen 1 (GRA1) induce cell death, which may help the parasite to survive by killing the host’s immune cells (Tsunawaki et al., 1988; Seabra et al., 2004).

Another survival and pathogenic tactic of the infecting *E. histolytica* is to inhibit the respiratory burst (ROS: H_2_O_2_, ${\text{O}}_{2}^{-}$, OH^−^, and NO production) of polymorphonuclear leucocytes/macrophages by using iron-containing superoxide dismutase (FeSOD) and NADPH:flavin oxidoreductase to detoxify the ROS (Lo and Reeves, 1980; Arbo et al., 1990; Bruchhaus and Tannich, 1994). *Entamoeba histolytica* uses a 29 kDa surface protein, namely peroxiredoxin, to resist the phagocyte reactive oxygen system, particularly the highly toxic agent peroxynitrite (ONOO^−^) (Pacheco-Yepez et al., 2014).

### Evasion of Complement-Mediated Killing

Parasites have evolved several strategies to escape complement-mediated killing [see the review by (Shao et al. (2019)], including the expression of orthologs of human proteins or acquisition of the host complement regulatory proteins onto the parasite surface to inhibit complement activation, and/or the expression of parasite proteins that bind to complement components to inhibit the complement protein functions.

The human complement system preys on the infecting *T. cruzi* (Lidani et al., 2017). The molecular factors (both host- and parasite-derived) and mechanisms used by *T. cruzi* to evade the host complement system have been previously reviewed (Ramirez-Toloza and Ferreira, 2017). *Trypanosoma cruzi* as well as the nematodes *Tichinella spiralis* and *Brugia malayi* evade the human complement system by producing homologs of the vertebrate calreticulin (TcCRT, TsCRT, BmCRT, respectively), which bind to the collagen-like triple-helical structures of C1q complement protein; thus, inhibiting the formation of the C1 complex (C1qC1r_2_C1s_2_), and the generation of the products of the complement classical pathway, including opsonins, chemotactic factors, and membrane attack complexes (MACs) (Ferreira et al., 2004; Aguilla et al., 2005; Valck et al., 2010; Yadav et al., 2017; Zhao et al., 2017). Besides, Clq captured by TcCRT on the parasite surface is recognized by the CD91 on the host cells; this facilitates the cell internalization of the parasite [CD91 is also called α2-macroglobulin receptor or a low-density lipoprotein receptor-related protein 1 (LRP1), which is a receptor for heat shock protein gp96] (Aguilla et al., 2005). *Trypanosoma cruzi* calreticulin binds to ficolins (L-ficolin or ficolin 2 and H-ficolin or ficolin 3) and mannose-binding lectin (MBL) in human serum and inhibits the lectin pathway for complement activation (Kahn et al., 1996; Sosoniuk et al., 2014). The *T. cruzi* metacycling trypomastigotes (the human infective stage) express the hemolytically inactive fragment iC3b, which cannot participate in C5 convertase formation; and thus, it is unable to form the lytic C5b-9 complex (MAC) (Joiner et al., 1986). *Trypanosoma cruzi* produces also complement regulatory protein (TcCRP) or gp160 (Norris et al., 1989; Norris and Schrimpf, 1994), complement C2 receptor inhibitor trispanning (TcCRIT) (Cestari et al., 2008), gp58/68 (C3 convertase inhibitor assembled by preventing the binding of Factor B to surface-fixed C3b in the alternate pathway) (Fischer et al., 1988), and decay accelerating factor (DAF) (Tambourgi et al., 1995; Shao et al., 2019), to similarly inhibit the complement activation, but by different mechanisms at different steps of the complement cascades. The microvesicles secreted by *T. cruzi* and also those derived from the host cells (induced by the *T. cruzi* metacyclic trypomastigotes) interact with C3 convertase; which thus inhibits complement activation, as well as increases the host cell invasion and parasite survival (Cestari et al., 2012; Ramirez et al., 2016; Wyllie and Ramirez, 2017; Shao et al., 2019).

*Schistosoma* spp. and *Taenia solium* use the paramyosin (a 94/97 kDa surface protein with an immunomodulatory property) that they produce to inhibit complement C1 (Laclette et al., 1992; Parizade et al., 1994) and to bind to C8 and C9 to inhibit MAC formation (Deng et al., 2003). The trophozoites of pathogenic *E. histolytica* shed C3 products from their surface by means of intact membrane motility (the capacity to release surface-bound complement proteins), which is one of the parasite’s complement-resistance mechanisms (Hamelmann et al., 1993). Moreover, the trypsin-sensitive membrane factor(s) of the *E. histolytica* trophozoites can interfere with the alternative pathway of complement activation, as it was revealed that treatment of the trophozoites with trypsin could reverse the parasite to complement lysis sensitivity (Hamelmann et al., 1993). *Leishmania* spp. expresses major surface protease (MSP) [alternately called gp63, leishmanolysin, EC3.4.24.36, and promastigote surface antigen (PSA), which is the most abundant surface protein of the *Leishmania* promastigotes] to resist complement-mediated lysis, and enhances the cellular entry of the *Leishmania* by a receptor-mediated mechanism (Yao et al., 2003). *Taenia taeniaeformis* cysts release molecules that are functionally analogous to cobra venom factor and polyanionic proteolytic proteoglycan, which activate complements away from their tegumental membrane; thus, blocking activation of either the alternative or classical complement pathways on the parasite surface. The complement fixation distant from the parasite membranes aids the parasite survival (Hammerberg et al., 1976; Leid, 1977; Hammerberg and Williams, 1978a; Hammerberg and Williams, 1978b).

### B Cells’ Manipulation by Parasites

B cells play an important role in immunity against parasitic infections, including in the production of antibodies, cytokines, and chemokines, and serve also as antigen-presenting cells that induce the parasite-directed T-cell responses. The B-cell responses during protozoan parasite infections have been previously reviewed (Vesely et al., 2012). Nevertheless, parasites have evolved strategies to evade B-cell immunity by interfering with B-cell development, inducing B-cell death, and stimulating polyclonal B cells to dilute the effects of a specific response, and may also inflict autoimmunity, as well as interfering with the memory B-cell response (Vesely et al., 2012).

*Trypanosoma cruzi* infection subverts B-cell generation by induction of the apoptosis of immature B cells in the bone marrow. This apoptosis was shown not to be *via* a Fas/FasL-dependent pathway, but instead was due to the CD11b^+^ myeloid cells in the bone marrow, which secrete a soluble factor, *i.e*., prostaglandin E2 (a lipid metabolite of the cyclooxygenase pathway), to deplete the immature B cells, which consequently reduces peripheral B-cell replenishment (Brown et al., 1992; Zuniga et al., 2005). Radwanska et al. (2008) demonstrated that *T. brucei* caused the loss of the IgM^+^ marginal zone (IgM^+^MZ) B-cell population, and hence disabled the hosts’ capacity to raise a long-lasting specific protective anti-parasite antibody response, as well as abrogated the vaccine-induced protective response to non-related human pathogens, such as *Bordetella pertussis* (Radwanska et al., 2008). *T. brucei* infection not only causes a depletion of the common lymphoid progenitors, *i.e*., pre-pro-B, pro-B, pre-B, and immature B cells in bone marrow, but also the death of splenic transitional B cells (both T1 and T2), by a mechanism that differs from the Fas/FasL-independent and prostaglandin-dependent death pathways caused by *T. cruzi.* Rather the *T. brucei-*mediated B-cell killing was shown to be parasite-B cell contact dependent (Magez et al., 2011). It was speculated that the densely packed VSG molecules on the surface of the trypomastigotes might cause B-cell receptor (BCR) clustering on the transitional B-cell surface, which would lead to hyperstimulation of the B cells; thus causing Fas/FasL-independent cell death, even in the absence of T-cell signaling (Magez et al., 2011). The death of the immature B cells should lead to the loss of the host’s antibody responses against the parasite as well as abrogation of the respective memory B-cell generation.

Individuals from malaria-endemic areas with a memory response against *Plasmodium* were found to have atypical memory B cells that expressed the transcription factor T-bet in their circulation (Weiss et al., 2009; Guthmiller et al., 2017; Obeng-Adjei et al., 2017). These cells also have inhibitory phenotypic markers, such as FcRL5, which delineates functionally impaired memory B cells on their surface (Sullivan et al., 2015). The atypical FcRL5^+^T-bet^+^ B cells were found to be expanded greatly in acute malaria in the circulation of *P. falciparum*-infected returned travelers and this was correlated with both the development of anemia and the plasma anti-phosphatidylserine (PS) autoantibodies in these patients. The *P. falciparum*-infected erythrocytes activate the FcRL5^+^T-bet^+^ B cells to release the anti-PS autoantibodies, which then bind to uninfected erythrocytes and induce the erythrocyte clearance, leading to anemia (Fernandez-Arias et al., 2016; Rivera-Correa et al., 2019). Expansion of the atypical memory B cells might be due to the direct interaction of the cysteine-rich interdomain region 1 α (CIDR1α) of the erythrocyte membrane protein 1 (PfEMP1) with the B cells, which modulates the host B-cell activating factor (BAFF) signaling pathway and compromises the protective immune memory by interfering with the B-cell differentiation (Scholzen and Sauerwein, 2013).

DNA or oligodeoxynucleotides (ODNs) containing CpG motifs (CpG-ODN) derived from *Babesia bovis*, *T. cruzi*, and *T. brucei* stimulate the proliferation of naïve B cells *via* the Toll-like receptor (TLR) 9 (TLR-9) signaling pathway (Shoda et al., 2001). *Trypanosoma cruzi* induces a polyclonal lymphocyte response (Minoprio et al., 1986). The infecting *T. cruzi* secretes glutamate dehydrogenase (GDH), which causes polyclonal proliferation and a differentiation of naïve B cells (Montes et al., 2006). B-cell stimulation by *T. cruzi* is T-cell independent (Montes et al., 2006); instead, the GDH induces the CD11b^+^ myeloid cells to produce IL-6, IL-10, and BAFF, which act synergistically to induce B-cell expansion and differentiation into plasma cells that produce non-specific polyclonal antibodies and that cause hypergammaglobulinemia, as well as supporting maintenance of the memory B cells (Bernasconi et al., 2002; Montes et al., 2006). The polyclonal activation of B lymphocytes was also seen in *S. mansoni* infection (Lopes et al., 1990). These activated polyclonal B cells were found to produce anti-liver autoantibodies, anti-DNA, or lymphocyte-reactive alloantibodies, as well as the formation of circulating immune complexes in the infecting host (Fischer et al., 1981). Polyclonal lymphocyte activation and autoantibodies to ribonucleoproteins were found in the sera of patients infected with *L. donovani*, in which the antibodies contributed to the dysfunction of the host immune system (Argov et al., 1989). Polyclonal B-cell activation during the acute phase of Chagas disease caused by *T. cruzi* infection was found to be correlated with a high level of serum antibodies with a low parasite specificity and a delay in the protective immune response (Bryan et al., 2010). The reduction and the delay of the parasite specific antibody response inevitably results in an inefficient control of the infection; all of which facilitates the parasite survival and thus, a protracted infection. Patients with visceral leishmaniasis develop hypergammaglobulinemia and autoantibodies, including anti-nuclear antibodies (ANAs), anti-smooth muscle antibodies (ASMAs), anti-dsDNA antibodies, IgM anti-cardiolipin antibodies, and IgM rheumatoid factor (RF), which resemble those of rheumatic autoimmune diseases, particularly systemic lupus erythematosus (SLE) (Voulgari et al., 2003; Ossadron et al., 2006; Sakkas et al., 2008).

### Modification of the Host Cell Activity and the Killing of Host Cells

*Entamoeba histolytica* secretes proteins to modulate tumor necrosis factor (TNF) production by inflammatory macrophages, both locally and systemically, which might be another strategy of the amoeba to antagonize the TNF autocrine or paracrine roles for inhibiting granuloma formation and dissemination of the amoeba to other tissues (Wang et al., 1992). *E. histolytica* trophozoites possess the ability to mediate contact-dependent host cell killing (Ravdin and Guerrant, 1980; Ravdin et al., 1980; Ralston and Petri, 2011). The trophozoites nibble on and consume a small part of the host cellular material (cytoplasm and mitochondria), which is termed “Amebic trogocytosis” (Ralston, 2015a; Ralston, 2015b). This causes the host cell to lose membrane integrity and the mitochondrial potential, ultimately bringing about the cell death. Trogocytosis caused by *E. histolytica* contributes to host cell killing and tissue invasion, especially during intestinal invasion (Ralston et al., 2014). Other pathogenic amoebae named *Naegleria fowleri* and *Balamuthia mandrillaris*, the causative agents of primary amebic meningoencephalitis (PAM) and granulomatous amebic encephalitis, respectively, are termed as brain-eating amebae, as they trogocytose brain cells (Fowler and Carter, 1965; Cooter, 2002; Shadrach et al., 2005). *N. fowleri* uses a specialized cytoplasmic extension called “Amebostome” or “Food-cup” for host cell attachment and ingestion (Grace et al., 2015). *Entamoeba histolytica* stimulates the chemotaxis of human polymorphonuclear neutrophils (PMNs) (Salata et al., 1989). It uses a pore-forming polypeptide, which is structurally similar to melittin (the membranolytic peptide of bee venom), to make amebic pores on the host cells; thereby causing host cell lysis (Leippe et al., 1991). It was estimated that one *E. histolytica* trophozoite can kill up to 3000 neutrophils (Guerrant et al., 1988). The host cell killing by the amebae is contact dependent and is mediated by an amebic galactose/N-acetylgalactosamine (Gal/GalNAc) adherence lectin that recognizes specific ligands on the target cells. The target cell death caused by *E. histolytica* may be necrotic (Beninghausen and Leippe, 1997) or caspase 3-dependent apoptotic (Huston et al., 2000). *E. histolytica* trophozoites can phagocytose the attached host cells, both living and the ameba-mediated apoptotic cells. The host cell phagocytosis is essential for amebic growth and pathogenesis. The phagocytic process involves continuous and distinct but coordinated steps, including recognition of the target cells and binding to cellular ligands using the parasite Gal/GalNAc-specific lectin, activation of a signaling pathway involving the actin-myosin IB cytoskeleton and PI3-kinase, leading to cytoskeletal reorganization, and vesicle trafficking (Marion et al., 2005; Okada and Nozaki, 2006). During phagocytosis, the amebic RAB5 plays an important role in the formation of unique vacuoles, which are essential for engulfment of the host cells. The *E. histolytica* RAB5 is also important for packaging of the lysosomal hydrolases prior to the phagosome–lysosome fusion (Saito-Nakano et al., 2004). The phosphatidylserine (PS) exposed on the apoptotic host cell surface facilitate *E. histolytica* engulfment of the cells (Bailey et al., 1987; Huston et al., 2003).

### Formation of Immune Complexes

In parasitic diseases, immune complexes can be formed at the site of parasite penetration, in the tissues, or in the circulation. Although most immune complexes can be removed by the normal physiological function of the host body, nevertheless, immune complexes formed between soluble antigens of some infecting parasites with the host’s circulating antibodies may bestow the parasites with the ability to avoid the host immunological attack *via* antibody-dependent cell-mediated cytotoxicity (ADCC), complement-mediated lysis, or antibody-mediated opsonization and phagocytosis, as well as causing immunopathology. For example, *Echinostoma caproni* (class Trematoda, family Echinostomatidae) produces excretory/secretory products that cover the parasite surface. The antibodies bound to these products, the so-formed immune complexes, are then degraded by the parasite-derived proteases, which is the parasite’s way to evade the antibody-mediated attack (Cortés et al., 2017). Microfilariae of the *B. malayi* filarial worm produce stage-specific proteins that form circulating immune complexes with the host antibodies in the plasma of the microfilaremic subjects. The antibodies to these proteins are not free in the microfilaremic sera, raising the possibility that the immune complex-forming proteins of the circulating microfilariae play a role in the immune-evasion mechanism of the circulating larvae to avoid antibody-mediated host immunity (Reamtong et al., 2019). Circulating immune complexes have been detected in the sera of patients with both localized and generalized onchocerciasis (river blindness caused by the filarial worm *Oncocerca volvulus*) (Paganelli et al., 1980). Circulating immune complexes have also been found to be formed in patients with schistosomiasis (Bout et al., 1977; Smith et al., 1977; Lapa et al., 2013).

### Modulation/Suppression of the Host Immune Responses

#### Modification of the Macrophage Functions

Macrophages are cells of the mononuclear phagocytic system (formerly the reticuloendothelial system) and play a role in the first line of defense against invading pathogens. These cells are classified into two main types: classically activated (M1) and alternatively activated (M2) macrophages (Mantovani et al., 2004; Zhang and Mosser, 2008; Murray, 2017; Yao et al., 2019). The M1 macrophages are activated after exposure to type 1/Th1 cytokines (such as IFN-γ, TNF-α), pathogen-associated molecular patterns (PAMPs), such as bacterial lipopolysaccharide (LPS), and damage-associated molecular patterns (DAMPs) (Zhang and Mosser, 2008; Murray, 2017; Yao et al., 2019). The M1 macrophages express TLR-2, TLR-4, CD80/CD86, inducible nitric oxide synthase (iNOS), and MHC class-II on their surface and produce pro-inflammatory cytokines (*e.g*., IL-1α, IL-1β, IL-6, IL-12, IL-18, and IL-23, TNF-α, and type I interferons) and many chemokines, such as CXCL9 and CXCL10, which induce a type 1/Th1 adaptive immune response (Mantovani et al., 2004; Zhang and Mosser, 2008; Murray, 2017; Atri et al., 2018; Yao et al., 2019; Lee et al., 2020). The M1 macrophages phagocytose pathogens, including parasites, bacteria, and viruses, and kill them using nitric oxide (NO) generated by the iNOS or reactive oxygen intermediates (ROI) from the NADPH oxidase of the respiratory burst system, and enzymes of the oxygen-independent pathway (Nagy and Haschemi, 2015; Atri et al., 2018). The alternatively activated (M2) macrophages express different phenotypes and functions in response to different environmental cytokine milieu. They are currently classified into 4 different subgroups: M2a, M2b, M2c, and M2d. M2a generated in response to IL-4 and IL-13 expressing IL-10, TGF-β, CCL17, CCL18, and CCL22. The M2a macrophages enhance phagocytosis and promote cell growth and tissue repair. Immune complexes (ICs), ligands of the Toll-like receptors (such as LPS), and IL-1 induce M2b macrophages to release TNF-α, IL-1β, IL-6, IL-10, and TGF-β. Glucocorticoids induce M2c macrophages, which are known as inactivated macrophages that secrete IL-10, TGF-β, CCL16, and CCL18 and play crucial roles in the phagocytosis of apoptotic cells. Toll-like receptor antagonists induce M2d macrophages, while IL-4 plus IL-13 and IL-10 induce both M2a and M2c (Mantovani et al., 2004; Yao et al., 2019). The M2 macrophages are distinct from the M1 macrophages in expressing high levels of arginine-metabolizing enzyme, arginase-1 (particularly M2a and M2c) (Mantovani et al., 2004; Yao et al., 2019), the chitinase-like molecule Chi3L3 (Ym1/ECF-L, which is an eosinophil chemotactic factor), the resistin-like molecule FIZZ1/RELM-α, and an acute-phase protein (serum amyloid A3) (Loke et al., 2002). After exposure to cytokines (such as IL-4, IL-10, IL-13, IL-33, TGF-β), IC, or apoptotic cells, the M2 macrophages produce arginase-1, which hydrolyzes L-arginine to urea and L-ornithine; here, L-ornithine can be further metabolized to the amino acid proline, which is required for collagen production and the development of fibrosis in tissue repair (Bronte and Zanovello, 2005; Raes et al., 2007). L-arginase inhibits the production of nitric oxide (NO) *via* several potential mechanisms, including competition with iNOS for L-arginine and the generation of scavengers for nitric oxide (NO), superoxide, and peroxynitrite (Durante et al., 2007). The M2 macrophages are anti-inflammatory and are associated with wound healing, matrix deposition, tissue repair/fibrosis/remodeling, immune regulation, immune suppression, allergy, and helminthic infections (Chen et al., 2019; Tang et al., 2019; Lee et al., 2020). The newly recognized M2d macrophages promote angiogenesis and tumor progression by producing IL-10 and vascular endothelial growth factors (VEGFs) (Murray, 2017). Macrophages play an important role in the host immune responses against both protozoal and helminthic infections (Raes et al., 2007; Reyes and Terrazas, 2007; Bai et al., 2011).

Many infecting parasites manipulate and influence macrophages for their permissive parasitism (Noël et al., 2004). In the early phase of *S. japonicum* infection, NO produced by M1 macrophages is cytotoxic to schistosomulae, and thus prevents their further development into adult parasites, thus hindering the subsequent formation of schistosomal egg-mediated hepatic fibrosis (granuloma formation) (Barron and Wynn, 2011; Zhu et al., 2014). At the later stage of the infection, however, arginase-1 expressed by the M2 macrophages promotes hepatic schistosomal fibrosis caused by the parasite eggs (Zhu et al., 2014). A schistosomal worm extract was found to stimulate the generation of the M1 macrophages at the initial phase of the fluke infection, whereas the secretory/excretory antigen from the parasite eggs preferentially promoted the M2-polarized phenotype during the chronic stage of infection (Zhu et al., 2014). *Schistosoma mansoni* produces hemozoin, which is not only a potent immunomodulatory, but also induces M2 macrophage development (Truscott et al., 2013). Infection by *Brugia malayi* can be manifested in two main clinical outcomes: asymptomatic, with the parasite’s first-stage larvae (L1) in the blood circulation called microfilaremia, and chronic pathology, *i.e.*, elephantiasis, caused by adult parasites that occlude the lymphatic circulation. *B. malayi* microfilariae induce the generation of monocytes with a regulatory phenotype, *i.e*., the expression of PD-L1 and IL-10, which suppress the T-cell functions typically seen in lymphatic filariasis (O’Regan et al., 2014). Protozoan infections, including *Leishmania*, *Trypanosoma*, and *Plasmodium*, influence disease progression (higher parasite growth and disease severity) through the generation of M2 macrophages (Vincendeau et al., 2003; Duleu et al., 2004; Stempin et al., 2004; Raes et al., 2007). In African trypanosomiasis, NO and prostaglandins secreted by suppressor macrophages inhibit the effective T-cell response (Schleifer and Mansfield, 1993). Human alveolar echinococcosis (AE) is a very rare but severe zoonotic helminthic disease caused by the slowly proliferative larvae (metacestode) of *Echinococcus multilocularis.* The infection is common in certain rural communities in China. Macrophages from *E. multilocularis*-infected mice were found to have an impaired ability to present an antigen to specific T cells, which triggered the unresponsiveness of T cells and suppressed their clonal expansion during the chronic phase of AE; this activity was attributed to the CD40 and B7 costimulators expressed on the macrophages (Mejri and Gottstein, 2006).

#### Manipulation of Dendritic Cells (DCs)

Dendritic cells (DCs) are accessory cells of the mammalian immune system. DCs are professional antigen-presenting cells. They may be regarded as “sentinels” in innate immunity against pathogens. DCs are classified into three main groups: monocyte-derived DCs (mDCs), conventional DCs (cDCs, which can be further divided into cDC1/BDCA3/CD141, and cDC2/BDCA1/CD1c), and plasmacytoid DCs (pDCs) (Collin and Bigley, 2018). Immature DCs capture microbes or foreign substances that invade the body and process them into antigenic peptides, while the cells themselves differentiate into mature DCs. The mature DCs assemble the processed peptides with MHC class-II molecules on their surface for presentation to naïve T cells in the peripheral lymphoid tissues to induce primary adaptive immune responses. Besides MHC class-II, mature DCs express several surface molecules required for proper antigen presentation to T cells, including CD80/CD86, CD40, CD54, and CD83. They also produce different cytokines that influence the type of cognate T-cell response, such as IL-12 and IFN-γ for a type 1/Th1 response, and IL-4 for a type 2/Th2 response (Guermonprez et al., 2002). Viruses or tumors induce the pDCs to produce the type 1 interferon (IFN-α), TNF, IL-6, and granzyme B; cDC1 produces IFN-γ, TNF-α, IL-12, CXCL9, and CXCL10 in response to pathogens (both intracellular and extracellular); while cCD2 produces IL-1, IL-8, IL-10, IL-12, IL-23, and TNF-α after being activated by parasites and allergens (Collin and Bigley, 2018). Immature DCs that have been modulated by immunosuppressive cytokines such as IL-10, IL-35, and TGF-β (the so-called tolerogenic DCs) play a critical role in the induction of peripheral tolerance by causing effector CD4^+^ and CD8^+^ T-cell anergy, or by secreting IL-10 and TGF-β to induce naïve T-cell differentiation into regulatory T cells (Tregs) (Guermonprez et al., 2002; Kuwana, 2002).

Both protozoa and helminths modulate DC responses or interfere with the DC activities to compromise the effective immune response (type 1/Th1) against them. The parasites or their products skew the immune response toward the ineffective type 2/Th2 or regulatory response for their survival (reviewed in Terrazas et al., 2010). In helminthic infections, the interaction of the parasite antigens with the DCs *via* pathogen-recognition receptors (*e.g.*, Toll-like receptors, TLRs) tends to downregulate the pro-inflammatory cytokine production and potentiate the DCs for a type 2/Th2 response (MacDonald and Maizels, 2008; Carvalho et al., 2009).

The antigen of *B. malayi* microfilariae interferes with the differentiation of monocytes to immature DCs and/or immature DCs to mature DCs. The microfilarial antigen also inhibits the DC production of IL-12, IL-10, and IFN-γ in response to heterologous antigens, *e.g*., *Staphylococcus aureus* (Semnani et al., 2001). Besides, the *B. malayi* microfilariae reduce the human DC capacity to activate CD4^+^ T cells, inhibit their ability to make IL-12 and IL-10, and induce cell death (Semnani et al., 2003; Semnani and Nutman, 2004; Semnani et al., 2008). The phosphorylcholine-containing glycoprotein, named ES-62, secreted by filarial parasites modulates the production of IL-12 from macrophages and DCs *via* the TLR4 signaling pathway (Goodrige et al., 2005). Excretory/secretory glycoproteins of *Nippostrongylus brazilienzis* (NES) drive a Th2 response by upregulating the DC markers (CD86 and OX40 ligand), which promotes the type 2/Th2 response (Balic et al., 2004). *Schistosoma mansoni* soluble egg antigens (SEAs) that contain glycosylated proteins and lipoconjugates internalized by human DCs *via* multiple C-type-lectin receptors (such as DC-SIGN, mannose receptor, macrophage galactose-type lectin receptor) influence the DCs to induce naïve T cells to develop a type 2/Th2 response (van Liempt et al., 2007). Dendritic cells exposed to the antigen of *E. granulosus* were found to express less CD80/CD86 and had a reduced IL-12p70 production; and when they were used as antigen-presenting cells to present antigen to naïve T cells, the cognate T cells developed into Th2 cells that secreted IL-4 (Riganò et al., 2007).

*Plasmodium* species can modify the DC activities by several mechanisms, including by decreasing the total DC numbers, altering the myeloid and plasmacytoid DC ratio (increasing the number of the latter), and inducing Tregs (Wykes et al., 2007; Terrazas et al., 2010). Plasmodial infection causes changes in the splenic DC ability to stimulate antigen-specific T cells (Sponaas et al., 2006). *Plasmodium falciparum* merozoites inhibit the pro-inflammatory cytokine production by DCs, and instead enhance the cells to produce IL-10 to create a permissive environment for the parasite (Mukherjee and Chauhan, 2008). The hemazoin of *P. falciparum* inhibits mDC maturation, as shown by a reduced expression of MHC class-II, CD80/CD86, CD40, CD54, and CD83 (Skorokhod et al., 2004; Skorokhod et al., 2005). Dendritic cells exposed to *P. falciparum* hemazoin (but not the infected erythrocyte membrane) had impaired function. The T cells activated by these DCs lacked B-cell-helper activities; whereby they failed to secrete cytokines and migrated to the B-cell area in lymphoid follicles, which resulted in the absence of B-cell responses to heterologous antigens (Millington et al., 2006). Dendritic cells that were exposed to the *T. gondii* antigen became paralyzed, *i.e*., they did not produce IL-12 in response to the microbial product; the paralysis in the IL-12 production was not due to IL-10 or lymphocyte suppression (Reis e Sousa et al., 1999). Low virulent strains of *T. gondii* were found to inhibit the migration and maturation of human DCs (Diana et al., 2004). The lysate of *T. gondii* tachyzoite triggered the production of the endogenous lipoxin A4, which suppressed IL-12 production by DCs (Machado et al., 2006). *Toxoplasma gondii* attracts the migration of immature DCs and exploits the cells using a Trojan horse strategy for parasite dissemination in the host, but does not induce DC activation (*i.e.*, suppress the cytokine effector function of the cells) (McKee et al., 2004; Bierly et al., 2008). *Giardia lamblia* inhibits IL-12 production by LPS-stimulated DCs *via* IL-10 and phosphoinositide 3-kinase-dependent pathways (Kamda and Singer, 2009).

Effective immunity to *Leishmania* infection depends on the type 1/Th1 response initiated by IL-12 cytokine from antigen-presenting cells (DCs) that induce naïve T cells to differentiate into IFN-γ-secreting Th1 cells (Muller et al., 1989). However, *Leishmania* spp. and their products regulate the immunity by means of several strategies (Rodrigues et al., 2016), one of which is by interfering with the DC maturation process and by modulating the DC activities (Terrazas et al., 2010). *Leishmania amazonensis*-infected mice had intrinsic defects at the level of DC activation, *i.e*., their DCs secreted lower levels of IL-1α and IL-1β. The infected mice were less potent in activating the IL-12p40-producing CD11c^hi^CD45RB^–^CD83^+^CD40^+^ DC subset, and preferentially activated the susceptible CD4^+^ T-cell phenotype, IFN-γ^lo^IL-10^hi^IL-17^hi^; indicating that this parasite interferes with innate and adaptive immunity *via* altering the DC activities (Xin et al., 2007). The presence of live or heat-killed *L. amazonensis* during DC differentiation in one study was found to cause a decrease in CD80/CD86 and CD1a on the cell surface, and a lower secretion of IL-6, as well as an impairment of the antigen-presenting activity. Live *L. amazonensis* parasites also abrogated full DC differentiation, which caused a delay in the immune response to favor the parasite’s establishment in the human host (Favali et al., 2007). Through activation of the MAPK/ERK pathway, *L. amazonensis* promastigotes and amastigotes induce alteration of the DC phenotype, as manifested by a reduced surface expression of CD40 and CD83 and IL-12p40 production (Boggiatto et al., 2009). During *L. donovani* infection, the number of splenic DCs was found to be increased, but they failed to migrate from the marginal zone (MZ) to the periarteriolar lymphoid sheath (T-cell-enriched area) due to a reduced CCR7 expression (the cells responded poorly to the chemotactic activity of the CCR7 ligands, CCL21 and CCL19) (Ato et al., 2002). The spatial segregation of the DCs and T cells resulted in poor Th1 generation, and contributed to visceral leishmaniasis development (Ato et al., 2002). Likewise, *L. major* uses lysophosphoglycan (LPG) to modulate the Langerhans cell phenotype and inhibits the cell migration; thus interfering with antigen presentation to the T cells (Ponte-Sucre et al., 2001). Excretory–secretory antigens of *L. donovani* and *L. major* caused a decreased expression of CD40, CD86, HLA-DR, and DC-SIGN on human mature DCs and suppressed the production of IL-12p70 by the cells, rendering the cells the inability to induce a type 1/Th1 response (Ravest et al., 2008). Immature bone marrow-derived dendritic cells infected with *L. mexicana* amastigotes did not develop into mature DCs or secrete IL-12 (Bennett et al., 2001).

#### Exploitation of Immune Checkpoints

Immune checkpoints are inhibitory receptors expressed on immune cells that are crucial for maintaining self-tolerance and regulating the length and magnitude of immune responses, such that the responses are not too great or too extensive so as to cause tissue damage (Wykes and Lewin, 2018). These cellular receptors include cytotoxic T-lymphocyte-associated antigen 4 (CTLA4), in which the ligands are CD80/CD86 (Andrews et al., 2019); programmed cell death protein 1 (PD-1), in which the ligands are PD-L1 or B7-H1 and PD-L2 or B7-DC (Andrews et al., 2019); T-cell immunoglobulin and mucin-domain-containing molecule 3 (TIM-3/HAVCR2), in which the soluble ligands are galectin 9 and high mobility group box 1 protein (HMGB1) and the cell surface ligands are ceacam-1 and phosphatidyl serine (Acharya et al., 2020); lymphocyte-activation gene 3 (LAG-3/CD223), in which the canonical ligand is MHC class-II on the antigen-presenting cells (Buisson and Triebel, 2003); T-cell immunoreceptor with the immunoglobulin and immunoreceptor tyrosine-based inhibitory motif domain (TIGIT), in which the ligands are CD155 (PVR) and CD112 (PVRL2, nectin-2) (Chauvin and Zarour, 2020); CD200, which interacts with CD200R/OX2R (Minas and Liversidge, 2006); and the herpesvirus entry mediator (HVEM/TNFRSF14/CD270), in which the ligands include two canonical TNF superfamily ligands: LIGHT/TNFSF14 and LTα/lymphotoxin-α (Mauri et al., 1998), and unconventional ligands of the Ig superfamily [B and T lymphocyte attenuator (BTLA), CD160, and the viral envelop protein of the herpes simplex virus envelope glycoprotein D] (Cheung et al., 2009).

Immune checkpoint blockades that benefit the pathogens, including parasites, viruses, and bacteria, have been previously reviewed (Wykes and Lewin, 2018). *Plasmodium falciparum* and *P. vivax* infections activate immune checkpoint molecules (Wykes and Lewin, 2018). *P. falciparum* induces the expression of PD-1 on the CD4^+^ and CD8^+^ T cells of recently infected subjects (Butler et al., 2011; Illingworth et al., 2013). Individuals with acute-phase *P. falciparum* and/or *P. vivax* infections were found to have Treg cells with an increased expression of CTLA4, OX40 (TNFRSF4), glucocorticoid-induced TNFR-related protein (GITR/TNFRSF18), and CD69 (Illingworth et al., 2013). Blockade of CTLA-4 and other inhibitory receptors, *e.g*., lymphocyte-activation gene-3 (LAG-3), programmed death-1 (PD-1), and T-cell immunoglobulin mucin-3 (TIM-3), improved T-cell responses to malaria parasites (Butler et al., 2011; Hafalla et al., 2012; Redmond et al., 2014; Costa et al., 2015; Goncalves-Lopes et al., 2016). These evidences suggest that the malaria parasites exploit the immune suppression by Tregs through checkpoint control for their pathogenesis. Blockade of the PD-1-PDL-1 pathway could rescue the dysfunctional CD8^+^ T cells that control *T. gondii* infection, indicating that *T. gondii* use the immune checkpoint-signaling pathway for immune evasion (Bhadra et al., 2011). The clinical manifestation caused by *E. granulosus* is like a slow-growing tumor that can invade nearby tissue, such as the lung and liver (Torgerson et al., 2010). At the chronic stage of human alveolar echinococcosis (AE), lymphocyte proliferation is inhibited *via* the overexpressed PD‐1/PD‐L1 pathway; here, blockade of the immune checkpoint pathway could restore the host immune responses that mediate immunological control of this metacestode proliferation (Wang and Gottstein, 2016; Wang et al., 2018).

#### Induction of the Regulatory Cells of the Immune System

The regulatory cells of the immune system include regulatory T cells (Tregs) and regulatory B cells (Bregs), besides the toterogenic dendritic cells (DCs) and the alternatively activated macrophages, mentioned previously. These cells play important roles in the control of the magnitude and length of the immune responses to foreign antigens and the maintenance of the immune system homeostasis and self-tolerance (Mizoguchi et al., 2000; Hu and Wan, 2010; Jang et al., 2013; Okeke et al., 2019).

##### Regulatory T Cells (Tregs)

Besides maintaining self-tolerance and immune dysregulation in the periphery, Tregs also suppress auto-reactive T cells that escape negative selection in the thymus to prevent autoimmunity (Sakaguchi et al., 1995; Xing and Hogquist, 2012; Pellerin et al., 2014). Tregs are classified into two main groups: natural/central Tregs (nTregs) and inducible/peripheral Tregs (iTregs). The nTregs (phenotype: CD4^+^CD25^+^CTLA-4^+^Foxp3^+^) are generated in the thymus during negative selection, whereas the iTregs are generated in the periphery (Abbas et al., 2013). It should be noted that the canonical transcription factor Foxp3 (forkhead box p3/winged helix) of Tregs is also transiently expressed in activated human effector T cells (Wang et al., 2007; Ziegler, 2007). Different types of iTreg cells have been recognized, including converted Foxp3^+^ Tregs; Tr1, which preferentially secretes IL-10 and low amounts of TGF-β; Th3, which preferentially secretes TGF-β; Tr35, which principally secretes IL-35; and regulatory Th17 (Treg17), which secretes IL-17, a high level of aryl hydrocarbon receptor (AhR), and IL-10, but produces much reduced levels of IL-22 (Roncarolo et al., 2006; Tak et al., 2006; Xing and Hogquist, 2012; Singh et al., 2013; Pellerin et al., 2014; Bellemore et al., 2015; Sharma and Kinsey, 2018). The Tregs kill or inhibit the functional activities of the target cells (such as effector T cells, antigen-presenting cells), either directly by cell–cell contact, by secreting soluble suppressive factors, and by competing for growth factors, or indirectly through manipulation of the antigen-presenting cells, such as dendritic cells (DCs), and by converting them into tolerogenic DCs (Tak et al., 2006; Sojka et al., 2008; Sharma and Kinsey, 2018). Tregs use their surface molecules, *e.g*., CTLA-4, LAG-3, FasL, PD1, cell-surface-bound TGF-β, to interact with the respective cognate ligands on the target cells and deliver suppressive signals/cytotoxic factors, such as cyclic adenosine monophosphate (cAMP), granzyme B, and the FasL-Fas apoptotic signal to the targets. The Treg ectonucleases, CD73/CD39, generate adenosine, which binds to the A2A receptor to down-modulate the target cells’ immune functions. Tregs also secrete immunosuppressive cytokines, including IL-10, IL-35, and TGF-β, to impair the target cell activities. Usually, the Tregs do not produce IL-2 (Inaba and Geiger, 2006), but they compete with naïve/effector T cells for the cytokine by using their high affinity IL-2Ra (CD25) for their growth and immunosuppressive cytokine production, which leads to naïve/effector T-cell death through being neglected (Barthlott et al., 2005). Regulatory T cells can suppress effector T cells indirectly by downregulating the costimulatory molecules CD80/CD86 and MHC molecules on dendritic cells (DCs) *via* CTLA-4, which weakens the T-cell-stimulating activity of the DCs; *i.e.*, the DCs become “Tolerogenic DCs” (Velavan and Ojurongbe, 2011). The generated tolerogenic DCs produce indoleamine 2,3 dioxygenase (IDO), which catabolizes tryptophan *via* the kynurenine pathway, leading to a reduced local tryptophan concentration, and an elevation of the immunomodulatory tryptophan metabolites, *e.g*., kynurenic acid, which contributes to the resolution of inflammation and establishment of an immunosuppressive environment (Wirthgen et al., 2018).

The roles of regulatory T cells in mediating immunosuppression in the human host during parasitic infections have been previously reviewed (Belkaid et al., 2006). Many parasites exploit the regulatory network of the host immune system to dampen the immune response against them to maintain successful parasitism (Yurchenko et al., 2006; Belkaid, 2007). *Leishmania* causes the CCR5-dependent homing of naturally occurring CD4^+^ regulatory T cells to the sites of the infection, which favors parasite persistence (Campanelli et al., 2006; Yurchenka et al., 2006; Belkaid, 2007). Other parasites that induce the generation of regulatory T cells include *Schistosoma* spp., *Plasmodium* spp., and intestinal parasites, such as *Anisakis simplex* (Herring worm, which causes severe abdominal cramp in infected subjects), and *Heligosomoides polygyrus* (previously named *Nematospiroides dubius*, which is a mouse nematode of the family *Trychostrongylidae*) (Cai et al., 2006; Kitagaki et al., 2006; Taylor et al., 2006; Belkaid, 2007; McSorley and Maizels, 2012; Wirthgen et al., 2018). *Trypanosoma cruzi* induces a larger proportion of the Foxp3^+^ T-cell population, which helps to control the inflammatory responses in the heart during Chagas disease (Mariano et al., 2008; de Araújo et al., 2011). *Trypanosoma congolense* infection was found to be expanded by the recruitment of natural Foxp3^+^ Tregs that produce IL-10 to limit the IFN-γ production by CD4^+^ and CD8^+^ effector T cells and downregulate M1 macrophage activation, resulting in reduced TNF-α production (Guilliams et al., 2007).

Chronic filarial infection is associated with host immune modulation *via* Tregs, particularly in the microfilaremics, as evidenced by the existence of multiple Treg subsets, including Foxp3-expressing CD4^+^ cells that express TGF-β (Th3) as well as Foxp3-negative CD4^+^ cells that express IL-10 (Tr1) (Babu et al., 2005; Taylor et al., 2005; Babu et al., 2006; Taylor et al., 2007; Metenou et al., 2010; Wammes et al., 2012; Metenou and Nutman, 2013). The Tregs and their suppressive cytokines could actively modulate the Th1-, Th2-, and Th17-mediated inflammatory response in the initial phase of the filarial infection as well as the B-cell response, which favors chronic infection (Babu et al., 2005; Mariano et al., 2008). Adult *Brugia malayi* and their microfilariae (L1) (McSorley et al., 2008), *Leishmania* spp. (Barral-Netto et al., 1992; Barral et al., 1993; Rodrigues et al., 1998; Wilson et al., 1998; Li et al., 1999)., *Trypanosoma cruzi* (Gazzinelli et al., 1992), and *Toxoplasma gondii* (Hunter et al., 1995), secrete the human TGF-β orthologue as a virulent factor to induce failure of the host effective immune responses against them. Antigen-specific Tr1 cells in *O. volvulus* infection display elevated amounts of CTLA-4 after stimulation, which mediates the inhibition of other T-cell functions (Satoguina et al., 2002).

##### Regulatory B Cells

Besides the production of antibodies, cytokines, and chemokines, and the presentation of antigens to T cells, B cells with an anti-inflammatory property (suppression of delayed hypersensitivity) were first recognized in 1974 (Katz et al., 1974; Neta and Salvin, 1974). B cells that regulate other immune cell functions and the suppression of inflammations (such as inflammatory bowel disease and chronic colitis experimental autoimmune encephalitis) were named regulatory B cells (Bregs) by Bhan and colleagues in 2000 (Mizoguchi et al., 1997; Abbas et al., 2013). A short history of the Bregs has been reviewed elsewhere (Mauri and Ehrenstein, 2007; Peng et al., 2018). Currently, both mouse and human Breg pools are known to consist of B cells with heterogeneous phenotypes and functional activities (Rosser and Mauri, 2015; Sarvaria et al., 2017; Wortel and Heidt, 2017; Wąsik et al., 2018). The recognized human Breg phenotypes and functions include: CD24^hi^CD27^+^ B10 cells (akin to CD5^+^CD1d^hi^ in mice), which are competent IL-10 producers found in blood circulation, and suppress effector CD4^+^ T cells, with monocyte and dendritic cell functions (Iwata et al., 2011; Rosser and Mauri, 2015); CD19^+^CD24^hi^CD38^hi^ transitional B cells, which are found in blood and at the site of inflammation; these cells induce and maintain iTreg but limit Th1 and Th17 cell differentiation (Flores-Borja et al., 2013), and suppress hepatitis B virus (HBV)-specific CD8^+^ T-cell responses in an IL-10-dependent manner (Blair et al., 2010; Bosma et al., 2012; Das et al., 2012; Flores-Borja et al., 2013); CD19^+^IgM^+^CD27^+^ memory and CD19^+^CD24^hi^CD38^hi^ transitional B-cell subsets in healthy human blood, which suppress the proliferation and IFN-γ production of CD3/CD28-stimulated autologous CD4^+^ T cells in a dose-dependent manner *via* IL-10 secretion, as well as cell–cell contact (likely mediated through CD80 and CD86) (Khoder et al., 2014); and CD19^+^CD24^hi^CD27^+^ plasmablasts found in human blood, which produce IL-10 and suppress effector CD4^+^ T cells and dendritic cells (Matsumoto et al., 2014).

The immune regulatory mechanisms mediated by the Bregs are mainly through their secreted immunosuppressive cytokines, including IL-10, IL-35, and TGF-β. The IL-10, TGF-β, and IL-35 of Breg cells suppress the differentiation of pro-inflammatory leukocytes, such as TNF-α-producing monocytes, IL-12-producing DCs, Th17 cells, Th1 cells, and cytotoxic CD8^+^ T cells (Mauri and Ehrenstein, 2007). Breg cells can also induce the differentiation of immunosuppressive T cells, *i.e*., Foxp3^+^ T cells and Tr1 cells. Bregs also support the maintenance of iNKT cells. CD73 and CD39 of Bregs generate adenosine in the presence of a substrate (Kaku et al., 2014). The molecular mechanisms of immune suppression mediated by Bregs include the induction of Tregs, suppression of effector CD4^+^ and CD8^+^ T cells, and the suppression of NK cells, NKT cells, monocytes, and DCs (Bankoti et al., 2012). Besides, Bregs can be self-inhibitory, *i.e*., Bregs can suppress pathogenic antibody production, either by a direct effect or by a secondary effect *via* the suppression of helper T cells (Le Texier et al., 2011).

Several parasites exploit Bregs to suppress the effective immune responses against them. For example, schistosome egg antigens, including the glycoprotein IPSE/alpha-1, trigger the development of Bregs (Haeberlein et al., 2017). Schistosomes induce regulatory CD1d B cells, which could inhibit allergic inflammation by producing IL-10 and the generation of Tregs (van der Vlugt et al., 2012). IgG4-producing B cells associated with a tolerance to filarial infections have been proposed to stem from IL-10-producing Bregs, and this is an indicator of immunosuppression (Adjobimey and Hoerauf, 2010; van de Veen et al., 2013). Patients with multiple sclerosis that were infected with helminths, including *Hymenolepis nana*, *Trichuris trichiura*, *Ascaris lumbricoides, Strongyloides stercolaris*, and *Enterobius vermicularis*, were found to have an increased production of B‐cell-derived IL‐10 and neurotrophic factors as part of the parasite’s regulation of the host immunity, which causes a beneficial spillover effect to alter the course of the autoimmune disorder (Correale et al., 2008). Regulatory B-cell induction by helminths confers also a spillover effect against allergic disease (Hussaarts et al., 2011).

## Conclusion and Future Perspective

Parasites are biologically and morphologically distinct; nevertheless, they all are endowed with strategies to evade a myriad of host innate and adaptive immunological factors, for establishment of their protracted parasitism. In this review, the intricate mechanisms at the molecular levels used by the parasites for overcoming/escaping the host defence are not given in details due to a broad range of the covered topics. For elaboration, the readers should see the respectively referred articles. The parasites’ immune evasion mechanisms can be categorized into several strategies as summarized in **Table 2**. These include the parasites’ ability to overcome the host’s physical and physiological barriers and to hide away from the host immune recognition by: covering themselves with the host factors (antigenic camouflage); inhabiting immunological privileged sites that are unreachable by the host immunological factors; expressing stage-specific antigenic profiles and variants, and by sharing their antigens with the host factors (antigenic mimicry) in order to evade the host immunity; destroying or overworking the host cells; antagonizing the host immunological factors or cellular functions; and by mediating the suppression of immune cell activities. On the flip side, the parasites’ molecular factors and mechanisms are potential targets of specific diagnostics, therapeutics, and vaccines against the invading entities and diseases caused by the respective parasites.

**Table 2:** Strategies used by parasites to overcome, evade, and suppress host immunity.

Evasion strategies	Evasion means	Example of parasites	Factor/molecule/structure/mechanism involved	Ref.
1. Overcoming the hosts’ physical and physiological barriers	Skin penetration	Hookworms (*Ancylostoma duodenale* and *Necator americanus*)	Larvae enter the host through minute breaks in the skin	(Beaver, 1956; Albanese et al., 2001; Conville and Berry hill, 2007)
		*Strongyloides stercoralis*	Urocanic acid, a histidine metabolite of human skin attracts the parasite lavae	(Safer et al., 2007)
		*Schistosoma* spp.	Digest human skin elastin by using chymotrypsin-like and trypsin-like proteases	(Salter et al., 2000)
	Vector and the vector’s salivary factors	*Plasmodium* spp.	Biting infected mosquito delivers anti-histamine, vasodialtors, anti-coagulants. platelet aggregation inhibitors, and immunomodulators	(Beier et al., 1991; Zheng et al., 2014; Gomes et al., 2016)
		*Trypanosoma brucei*	Biting Tsetse fly delivers salivary thromboregulatory compounds (5’-nucleotidase-releated apyrase, adenodine deaminase), Antigen 5, Gloss 2 peptide, and kinesin heavy chain 1 (TbKHC1)	(Caljon et al., 2009; Caljon et al., 2010; De Muylder et al., 2013; Bai et al., 2015)
	Vector-induced mechanical damage	*Trypanosoma cruzi*	Biting wound caused by triatomine bug	(Moncayo and Yanine, 2006)
	Mechanical strategies to avoid immune defense mechanisms, *e.g.*, rapid extracellular gliding motility, cell traversal motility, invasion motility	Phylum Apicomplexa, *e.g.*, *Plasmodium* spp., *Toxoplasma gondii*	Thromospondin-related anonymous protein (TRAP), perforin-like protein (PLP1), circumsporozoite protein (CSP)	(Vanderberg, 1974; King, 1988; Mota et al., 1999; Yuda and Ishino, 2004; Kolářová, 2007; Tavares et al., 2013; Risco-Castillo et al., 2015)
	Resistance to serum toxic molecule; serum high density lipoproteins; trypanosome lytic factors (TLFs)	*Trypanosoma brucei*	Serum resistance-associated protein (SRA)	(Thomson et al., 2009)
	Withstanding gastric acidity	*Giardia lamblia* *Entamoeba histolytica* *Cryptosporidium parvum*	Cysts; a significantly decreased gastric acidity and modifications of the normal microenvironmental conditions of the gastric mucosa	(Schofield et al., 1992; Striepen, 2013; Hemphill et al., 2019)
	Inhibit intestinal epithelial cell turnover	*Giardia lamblia*	Arginine deiminase	(Stadelmann et al., 2012)
	Breaching mucus layer	*Entamoeba histolytica*	Cysteine proteases and glycosidases	(Moncada et al., 2003; Lidell et al., 2006; García et al., 2015; Nakada-Tsukui and Nozaki, 2016)
	Digesting extracellular matrix: *e.g.*, collagen type IV and V as well as laminin and fibronectin	*Entamoeba histolytica*	Many kinds of proteases	(Schulte and Scholze, 1989; Nakada-Tsukui and Nozaki, 2016)
	Exploit bile acid and salt	*Echinococcus granulosus*	Bile acids and salts help the parasite eggs to hatch in the intestine to release oncospheres that pass through the portal and lymphatic vessels and reach the liver where they usually settle and develop as larvae (metacestodes or hydatid cysts). Occasionally, the oncosperes may settle in other tissues such as brain, lung, bone and form hydatid cysts therein	(Wen et al., 2019)
2. Sequestration in the host’s immunological privileged sites	Reside in red blood cells	*Plasmodium* spp. *Babesia* spp.	GPI anchor surface proteins. Microneme proteins, Rhoptry proteins, peripheral surface proteins to bind to receptors on red blood cells	(Miller, 1965; David et al., 1983; Allred and Al-Khedery, 2004; Bowen and Walker, 2005; Cowman and Crabb, 2006; Bloch et al., 2019)
	Sequestration	*Plasmodium* falciparum	*Plasmodium falciparum* erythrocyte membrane proteins (PfEMPs)	(Miller, 1965; David et al., 1983)
		*Babesia* spp.	Parasite-induced red blood cell membrane proteins	(Allred and Al-Khedery, 2004; Bloch et al., 2019)
	Reside in macrophage	*Leishmania* spp.	Metacyclic promastigotes bind/attach to the complement receptors (CR) 1, CR3 (Mac-1), fibronectin receptor, and the mannose-fucose receptor (MR) on the surface of macrophages. Virulent *L. infantum chagasi* binds complement receptor 3 (CR3) but does not trigger NADPH oxidase activation and subsequent respiratory burst. On contrary, MR ligation promote inflammatory responses. The avirulent *Leishmania* bind both CR3 and MR. These may explain why promastigotes of virulent *Leishmania* spp. avoid using the MR during their invasion of macrophages and survived intracellularly.	(Wright and Silverstein, 1983; Da Silva et al., 1989; Sehgal et al., 1993; Kane and Mosser, 2000; Linehan et al., 2000; Gupta et al., 2013)
	Nurse cell	*Trichinella* spp.	Complex parasite-induced host process that involve muscle cell response (de-differentiation, cell cycle re-entry and arrest) and satellite cell responses (activation, proliferation and differentiation)	(Wu et al., 2008a)
	Resides in hollow organs	*Taenia* spp. *Ascaris lumbricoides* *Opisthorchis viverrini*	Avoid effective serum IgM and IgG Anti-complemenatry activity of intestine Intestinal trefoil factor (ITF) produced by goblet cells Decay accelerating factor (DAF) produced by columnar epithelial cells	(Sun et al., 1999; Andoh et al., 2001)
3. Antigenic disguise	Merosome	*Plasmodium* spp.	Merozoites generated in liver cells released inside vesicles, called merosomes, into the liver sinusoid lumen. The merosomes do not express macrophage-recognition signal, such as phosphatidyl-serine, thus escape phagocytosis by kuffer cells and dendritic cells; they are carried to the lung vasculature where the merozoite cargo is released into blood stream.	(Sturn et al., 2006; Rénia and Goh, 2016; Melanie et al., 2019)
	Camouflage with host-like/host-derived molecules	*Schistosoma mansoni*	Erythrocyte antigens (Rhesus, M, N, S, and Duffy), complement proteins, integrins, CD44, collagen, immunoglobulins, MHC class-I, and β_2-_microglobulin,	(Clegg et al., 1971; Goldring et al., 1976; Kemp et al., 1977; Snary et al., 1980; Braschi and Wilson, 2006; Braschi et al., 2006)
		*Schistosoma japonicum* *S. mansoni*	Paramyosin (Sj97 and Sm97), Fc fragments of immunoglobulins and complements C1 and C9 proteins	(Laclette et al., 1992; Loukas et al., 2001; Deng et al., 2007)
		*Onchocerca volvulus*	Factor H	(Meri et al., 2002)
		*Trypanosoma cruzi*	Parasite uses trans-sialidases to transfer sialic acid from the host glycoconjugates to the parasite surface	(Nardy et al., 2016)
		*Echinococcus granulosus*	Host cells in the pericyst	(Golzari and Sokouti, 2014)
4. Parasites exist in different development forms	Different morphological forms/stages-specific excretory-secretory products	*Brugia malayi* *Schistosoma* spp. *Trypanosoma* spp. *Plasmodium* spp. and many others	Many parasite exists in different forms and shapes. They may express different sets of genes at a particular time which causes antigenic polymorphism	(Florens et al., 2002; Gryseels et al., 2006; Moreno and Geary, 2008; Fitzpatrick et al., 2009; Mitchell et al., 2012; McWilliam et al., 2013; Colley et al., 2014; Smit et al., 2015; Rénia and Goh, 2016; Reamtong et al., 2019)
5. Antigenic variation	Surface antigen variation	Trypanosomatids	Variant-specific glycoproteins	(Cross, 1975; Umekita et al., 1988; Umekita et al., 1997; Pays, 2006)
		*Plasmodium* spp.*Babesia* spp.	Antigenic variation of the relapse variants	(Cox, 1962; Brown and Brown, 1965; Hommel et al., 1983; al-Khedery et al., 1999; Winter et al., 2005; Recker et al., 2011; Rénia and Goh, 2016; Bloch et al., 2019)
6. Antigen capping	Get rid of immune complex	*Trypanosoma brucei*	Flagella pockets, endosomal proteins: RAB5 and RAB11	(Barry, 1979; Pal et al., 2003; Engstler et al., 2004)
		*Entamoeba histolytica*	RAB11	(Espinosa-Cantellano and Martínez-Palomo, 2000; Chávez-Munguía et al., 2012)
		*Lesihmania donovani*	Stage-specific antibodies to amastigotes/promastigotes cause the parasite surface membrane antigens to aggregate, move along the longitudinal cell axis, form polar cell caps, and subsequently disappear	(Dwyer, 1976)
7. Molecular mimicry	Complement resistance	*Schistosoma mansoni* *Schistosoma haematobium*	Complement C2 receptor inhibitory trispannin (CRIT)	(Inal, 1999; Deng et al., 2003)
		*Brugia malayi*	Keratinocytes periphilin-1 like protein	(Kazerounian and Aho, 2003)
		*Plasmodium falciparum*	*Plasmodium falciparum* erythrocyte membrane protein 1 (PfEMP1)	(Ludin et al., 2011)
	Blood group-like antigens	*Schistosoma japonicum*	Blood group A, B, and O antigens	(Matsuse, 1956)
		*Fasciola hepatica*	Blood group A, H, and Lewis (Le) antigens	(Ben-Ismail et al., 1982)
		*Ascaris lumbricoides* var *suum*,	A- and B- like blood group antigens	(Oliver-Gonzalez, 1944)
		Hookworms		(Ota et al., 1954; Ota and Tadokoro, 1954)
		*Trichinella spiralis*	Forssman antigen	(Mauss, 1941)
		*Echinococcus granulosus*	Blood group P1 substance	(Levine et al., 1958)
	Thrombospondin-mimic region	*Plasmodium falciparum*	Circumsporozoite protein (CSP)	(Robson et al., 1988)
	Myocardial cell protein mimicry	*Trypanosoma cruzi*	Parasite proteins that mimic myocardial cell proteins and induce autoimmunity	(Tanowitz et al., 2009)
8. Modify and/or suppress the host immunological factors/immune responses				
8.1 Digestion of the host matrices, antimicrobial peptides, antibodies, and/or inhibition of the host factors	Digest extracellular matrix proteins	*Entamoeba histolytica*	Membrane-bound cysteine proteases	(Que and Reed, 2000)
	Cleavage intestinal antimicrobial peptides	*Entamoeba histolytica*	Cysteine proteases	(Que and Reed, 2000)
	Digest human IgG	*Entamoeba histolytica*	Cysteine proteases	(Que and Reed, 2000)
		*Trypanosoma cruzi*	Cysteine protease, Cruzipain	(Berasain et al., 2003)
		*Schistosoma mansoni*	pH and temperature-dependent trypsin-like endoprotease and metalloaminopeptidase Cercarial and schistosomular extracts	(Auriault et al., 1981; Pleass et al., 2000)
	Protease inhibitor	*Schistosoma mansoni*	Kunitz-type protease inhibitor (SmKI-1)	(Ranasinghe et al., 2015)
8.2 Resist killing by the host cells	Interfere phagocytic activity Reduce/inhibit oxidative radical production Resistance the highly toxic oxidative radicals	*Plasmodium* spp.	Hemozoin	(Belachew, 2018)
		Family Trypanosomatidae	Acid phosphatase (ACP)	(Baghaei, 2003; Navabi and Soleimanifard, 2015)
		*Leishmania donovani*	*Leishmania donovani* chemotactic factor (LCF)	(Wenzel and Van Zandbergen, 2009)
		*Trypanosoma cruzi*	A complex network of anti-oxidant enzymes: peroxidases (APX, CPX, and MPX), catalase, trypanothione reductase, and superoxide dismutases (SOD)	(Finzi et al., 2004; Piñeyro et al., 2011; Freire et al., 2017; Beltran-Hortelano et al., 2017)
		*Toxoplasma gondii*	Cytosolic and mitochondrial superoxide dismutases, peroxiredoxins, glutathione-S-transferase, glutaredoxin, catalase, and thioredoxin reductase Programmed cell death 5 (TgPDCD5) and dense granule antigen 1 (GRA1) induce cell death	(Ding et al., 2004; Seabra et al., 2004; Xue et al., 2017)
		*Entamoeba histolytica*	Iron-containing superoxide dismutase (FeSOD, NADPH:flavin oxidoreductase	(Lo and Reeves, 1980; Arbo et al., 1990; Bruchhaus and Tannich, 1994; Pacheco-Yepez et al., 2014)
	Neutrophil extracellular trap (NET) resistance	*Leishmania infantum, L. major*	3′-nucleotidase/nuclease (3′NT/NU)	(Guimarães-Costa et al., 2014)
		*Leishmania donovani*	Lipophosphoglycan (LPG)	(Chang and Dwyer, 1976; Holm et al., 2001; Gabriel et al., 2010)
		*Leishmania* spp.	Endonuclease (Lundep) in sand fly saliva	(Chagas et al., 2014)
	Inhibits phagosome maturation	*Leishmania donovani*	Lipophosphoglycan (LPG), Synaptotagmin V	(Chang and Dwyer, 1976; Holm et al., 2001; Vinet et al., 2009; Gabriel et al., 2010)
	Dilute the detrimental effect of oxidative radicals	*Leishmania amazonensis, L. mexicana*	Parasitophorous vacuoles	(Wilson et al., 2008)
	Phagosomal membrane rupture	*Trypanosoma cruzi*	Trypanosome trans-sialidase	(Schenkman et al., 1992; Schenkman and Eichinger, 1993)
	ROS promote parasite growth	*Trypanosoma cruzi*	Trypanosome trans-sialidase causes phagosome rupture, facilitating the parasite accessibility to iron in cytoplasm	(Cardoso et al., 2016)
	Avoid cellular autophagy	*Toxoplasma gondii*	Micronemal proteins containing epidermal growth factor (EGF)-like domains, TgMICs: TgMIC3, TgMIC6	(Meissner et al., 2002; Wang et al., 2009; Muniz-Feliciano et al., 2013)
	Killing host immune cells	*Toxoplasma gondii*	*T. gondii-*programmed cell death 5 (TgPDCD5) and dense granule antigen 1 (GRA1)	(Tsunawaki et al., 1988; Seabra et al., 2004)
8.3 Evasion of complement-mediated killing	Interfering classical pathway	*Trypanosoma cruzi, Trichinella spiralis, Brugia malayi*	Homologs of the vertebrate calreticulin; TcCRT, TsCRT, BmCRT, respectively	(Ferreira et al., 2004; Yadav et al., 2017)
		*Trypanosoma cruzi*	Complement regulatory protein (TcCRP) or gp160 Complement C2 receptor inhibitory trispanning (TcCRIT)	(Norris et al., 1989; Norris and Schrimpf, 1994; Cestari et al., 2008)
		*Schistosoma* spp., *Taenia solium*	Paramyosin (a 94/97 kDa surface protein with an immunomodulatory property) that the parasites produce to inhibit complement C1	(Laclette et al., 1992; Parizade et al., 1994)
		*Taenia taeniaformis*	Analogous molecules of cobra venom factor, polyanionic proteolytic proteoglycan	(Hammerberg et al., 1976; Leid, 1977; Hammerberg and Williams, 1978a; Hammerberg and Williams, 1978b)
	Interfering lectin pathway	*Trypanosoma cruzi*	Calreticulin; Complement C2 receptor inhibitory trispanning (TcCRIT)	(Kahn et al., 1996; Cestari et al., 2008; Sosoniuk et al., 2014)
	Interfering alternative pathway	*Trypanosoma cruzi*	Complement regulatory protein (TcCRP) or gp160, gp58/68	(Fischer et al., 1988; Norris et al., 1989; Norris and Schrimpf, 1994)
		*Entamoeba histolytica*	Trypsin-sensitive membrane factor(s)	(Hamelmann et al., 1993)
		*Taenia taeniaformis*	Analogous molecules of cobra venom factor, polyanionic proteolytic proteoglycan	(Hammerberg et al., 1976; Leid, 1977; Hammerberg and Williams, 1978a; Hammerberg and Williams, 1978b)
	Inhibit complement amplification loop (Terminal pathway)	*Trypanosoma cruzi*	Hemolytically inactive fragment iC3b, Decay accelerating factor (DAF), Microvesicles	(Norris et al., 1989; Tambourgi et al., 1995; Cestari et al., 2012; Ramirez et al., 2016; Wyllie and Ramirez, 2017; Shao et al., 2019)
	Shed C3 products	*Entamoeba histolytica*	Surface-bound complement proteins	(Hamelmann et al., 1993)
	Resistance to complement-mediated lysis	*Leishmania* spp.	Major surface protease (MSP), alternately called gp63, leishmanolysin, EC3.4.24.36, and promastigote surface antigen (PSA)	(Yao et al., 2003)
8.4 B cells’ manipulations by parasites	B cell killing	*Trypanosoma cruzi*	Induction of the apoptosis of immature B cells in the bone marrow *via* prostaglandin E2 from CD11b^+^ myeloid cells	(Brown et al., 1992; Zuniga et al., 2005)
		*Trypanosoma brucei*	VSG molecules	(Magez et al., 2011)
	Generation of atypical memory B cells	*Plasmodium* spp.	Cysteine-rich interdomain region 1α (CIDR1α) of the erythrocyte membrane protein 1 (PfEMP1)	(Weiss et al., 2009; Scholzen and Sauerwein, 2013; Fernandez-Arias et al., 2016; Guthmiller et al., 2017; Obeng-Adjei et al., 2017; Rivera-Correa et al., 2019)
	Non-specific polyclonal antibody production: dilution of effective antibody, causing auto-immunity or cancer	*Babesia bovis* *Leishmania* spp *Schistosoma haematobium* *Trypanosoma cruzi* *Trypanosoma brucei*	DNA or oligodeoxynucleotides (ODNs) containing CpG motifs (CpG-ODN), Glutamate dehydrogenase (GDH)	(Fischer et al., 1981; Argov et al., 1989; Shoda et al., 2001; Bernasconi et al., 2002; Voulgari et al., 2003; Montes et al., 2006; Ossadron et al., 2006; Sakkas et al., 2008; Bryan et al., 2010)
8.5 Modification of the host cell activity and host cell killing	Trogocytosis	*Entamoeba histolytica* *Naegleria fowler* *Balamuthia mandrillaris*	Trophozoites possess the ability to mediate contact-dependent host cell killing Amoebostome or food-cup	(Fowler and Carter, 1965; Ravdin and Guerrant, 1980; Ravdin et al., 1980; Cooter, 2002; Shadrach et al., 2005; Ralston and Petri, 2011; Ralston et al., 2014; Ralston, 2015a; Ralston, 2015b)

	Pore forming	*E. histolytica*	Pore-forming polypeptide, amebic pores	(Salata et al., 1989)
	Phagocytosis	*E. histolytica*	Gal/GalNAc-specific lectin, amebic RAB5	(Saito-Nakano et al., 2004; Marion et al., 2005; Okada and Nozaki, 2006)
8.6 Immune complex formation	Avoid antibody-dependent cell mediated cytotoxicity (ADCC), complement-mediated lysis, antibody-mediated opsonization, phagocytisis	*Echinostoma caproni*	Excretory/secretory products, parasite-derived protease	(Cortés et al., 2017)
		*Brugia malayi*	Immune complex forming proteins from microfilariae in blood circulation	(Reamtong et al., 2019)
		*Onchocerca volvulus* *Schistosoma* spp.	Circulation immune complexes	(Bout et al., 1977; Smith et al., 1977; Paganelli et al., 1980; Lapa et al., 2013)
8.7 Modulation/suppression of the host immune responses				
8.7.1 Modification of the macrophage function	M1 Macrophage polarization	*Schistosoma* spp.	Schistosomal extract	(Zhu et al., 2014)
	M2 Macrophage polarization	*Schistosoma* spp.	Secretory/excretory antigen from eggs	(Zhu et al., 2014)
		*Schistosoma mansoni*	Hemozoin	(Truscott et al., 2013)
		*Leishmania* spp. *Trypanosoma* spp. *Plasmodium* spp.	The protozoan infections	(Vincendeau et al., 2003; Duleu et al., 2004; Stempin et al., 2004; Raes et al., 2007)
	Regulatory phenotype of monocytes/suppressor macrophages	*Brugia malayi*	Microfilarial (Mf) lysate	(O’Regan et al., 2014)
	Macrophage activation	*Trypanosoma brucei rhodesience*	The parasite induces the release of reactive nitrogen intermediates and prostaglandin, which down-regulate proliferative responses by T cells during infection	(Schleifer and Mansfield, 1993)
	Antigen presentation impairment	*Echinococcus multilocularis*	The parasite affects CD40 and B7 costimulator expression on peritoneal macrophages of infected mice and impairs peritoneal T cell activation	(Mejri and Gottstein, 2006)
8.7.2 Manipulation of dendritic cells	Inhibit dendritic cell (DC) production	*Brugia malayi* *Plasmodium* spp.	Microfilarial antigen Merozoites	(Semnani et al., 2001; Wykes et al., 2007; Mukherjee and Chauhan, 2008; Terrazas et al., 2010)
	Induce type 2/Th2 response	*Nippostrongylus brazilienzis*	Excretory/secretory glycoproteins	(Balic et al., 2004)
		*Schistosoma mansoni*	Soluble egg antigens (SEAs)	(van Liempt et al., 2007)
		*Ecchinococcus granulosus*	The parasite antigen B impairs human dendritic cell differentiation and polarizes immature dendritic cell maturation towards a Th2 cell response	(Riganò et al., 2007)
	Enhance IL-10 production	*Plasmodium falciparum*	Phagocytosed-free merozoites prevented soluble CD40 ligand-induced, phenotypic maturation of DCs and secretion of IL-12p70 but enhanced IL-10 production and primed, naive CD4^+^ cells to produce a high level of IL-10 compared with IFN-γ	(Mukherjee and Chauhan, 2008)
	Inhibit DC maturation	*Plasmodium* spp.	Hemazoin (malaria pigment)	(Bennett et al., 2001; Skorokhod et al., 2004; Diana et al., 2004; Skorokhod et al., 2005; MacDonald and Maizels, 2008)
		*Toxoplasma gondii*	The parasite products	
		*Leishmania mexicana*	Amastigote	
	Impaired DC function	*Brugia malayi*	ES-62	(Semnani et al., 2001; Semnani et al., 2003; Semnani et al., 2008; Semnani and Nutman, 2004; Goodrige et al., 2005; Sponaas et al., 2006)
		*Plasmodium* spp.	Merozoite surface protein 1 (MSP1)	
		*Plasmodium falciparum*	Hemazoin	(Millington et al., 2006)
		*Toxoplasma gondii*	Rhoptry (ROP) and dense granule (GRA) effector proteins	(Reis e Sousa et al., 1999; McKee et al., 2004; Machado et al., 2006)
		*Giardia lamblia*	Parasite extracts enhance DC to secrete IL-10 but reduced secretion of IL-12. The cells also had reduced expression of MHC class II, CD80, and CD86	(Kamda and Singer, 2009)
		*Leishmania amazonensis*	Live *L. amazonensis* parasites, but not of soluble *Leishmania* antigen. The parasite caused a decrease in CD80 and CD1a expression and an increase in CD86, decreased IL-6 production	(Xin et al., 2007; Favali et al., 2007)
		*Leishmania donovani, L. major*	Excretory-secretory antigens	(Ravest et al., 2008)
	Alteration of DC phenotype	*Leishmania amazonensis*	Promastigotes and amastigotes	(Boggiatto et al., 2009)
	Inhibit DC migration	*Leishmania donovani*, *L. major*	Lysophosphoglycan. (LPG)	(Ponte-Sucre et al., 2001; Ato et al., 2002)
8.7.3 Exploitation of immune checkpoints	Activate immune checkpoint molecules PD1, PDL1, CTLA4, LAG-3 and TIM3	*Plasmodium falciparum* *P. vivax*	Merozoites	(Butler et al., 2011; Hafalla et al., 2012; Illingworth et al., 2013; Redmond et al., 2014; Costa et al., 2015; Goncalves-Lopes et al., 2016; Wykes and Lewin, 2018)
		*Toxoplasma gondii* *Echinococcus granulosus*	The parasite components and molecular mechanisms are not elucidated	(Bhadra et al., 2011; Wang and Gottstein, 2016; Wang et al., 2018)
8.7.4 Induction of the regulatory cells of the immune system				
8.7.4.1 Regulatory T cells (Tregs)	Recruitment of natural occurring Tregs (nTregs)	*Leishmania* spp. *Trypanosoma congolense*	The parasite components and molecular mechanisms are not elucidated	(Campanelli et al., 2006; Belkaid, 2007; Guilliams et al., 2007)
	Induce the generation of inducible Tregs (iTregs)	*Schistosoma* spp. *Plasmodium* spp. *Anisakis simplex* *Heligosomoides polygyrus* *Trypanosoma cruzi*	The parasite components and molecular mechanisms are not elucidated	( Cai et al., 2006; Kitagaki et al., 2006; Belkaid, 2007; Mariano et al., 2008; de Araújo et al., 2011; McSorley and Maizels, 2012; Wirthgen et al., 2018)
	Induce the generation of Tr1	*Onchocerca volvulus*	The generated Tr1 cells display elevated amounts of CTLA-4 after stimulation, which mediates the inhibition of other T-cell functions	(Satoguina et al., 2002; Taylor et al., 2006)
	Induce the generation of Tr1, Th3	*Brugia malayi*	TGF-beta homolog (TGH) from both adults and microfilariae	(Babu et al., 2005; Taylor et al., 2005; Babu et al., 2006; Taylor et al., 2007; Metenou et al., 2010; Wammes et al., 2012; Metenou and Nutman, 2013)
		*Toxoplasma gondii* *Leishmania* spp. *Trypanosoma cruzi*	TGF-beta homolog (TGH)	(Barral-Netto et al., 1992; Gazzinelli et al., 1992; Barral et al., 1993; Hunter et al., 1995; Rodrigues et al., 1998; Wilson et al., 1998; Li et al., 1999; McSorley et al., 2008)
8.7.4.2 Regulatory B cells (Bregs)	Induction of various Bregs development, *e.g.*, IL-10-producing CD1d^high^CD5^+^ regulatory B cells, B10	*Schistosoma* spp.	Schistosome egg antigen, glycoprotein IPSE/alpha-1	(Correale et al., 2008; van der Vlugt et al., 2012, Haeberlein et al., 2017)
		Human filaria	Possibly the IgG4-producing B cells induced by the parasite infection	(van de Veen et al., 2013)
		*Hymenolepis nana* *Trichuris trichiura* *Ascaris lumbricoides* *Strongyloides stercoralis* *Enterobius vermucularis* *Toxoplasma gondii*	The parasite infected subjects were found to have an increased production of B‐cell-derived IL‐10 and neurotrophic factors although the parasite components are not elucidated	(Jeong et al., 2016; Haeberlein et al., 2017)

## Author Contributions

Both authors compiled and analyzed the information and wrote and edit the manuscript. All authors contributed to the article and approved the submitted version.

## Funding

The authors acknowledge and thank the Faculty of Medicine Siriraj Hospital, Mahidol University, for English editing and publication costs.

## Conflict of Interest

The authors declare that this review article was conducted in the absence of any commercial or financial relationships that could be construed as a potential conflict of interest.

## Publisher’s Note

All claims expressed in this article are solely those of the authors and do not necessarily represent those of their affiliated organizations, or those of the publisher, the editors and the reviewers. Any product that may be evaluated in this article, or claim that may be made by its manufacturer, is not guaranteed or endorsed by the publisher.
